# Supplementary material for: Differential impact of ubiquitous and muscle dynamin 2 isoforms in muscle physiology and centronuclear myopathy
Source: Nat Commun. 2022 Nov 11;13:6849. doi: 10.1038/s41467-022-34490-4 (PMC9652393; doi:10.1038/s41467-022-34490-4)
Supplement: Supplementary file 1 — Supplementary Information [file 41467_2022_34490_MOESM1_ESM.pdf]

Supplementary Figures

Supplementary Figure 1

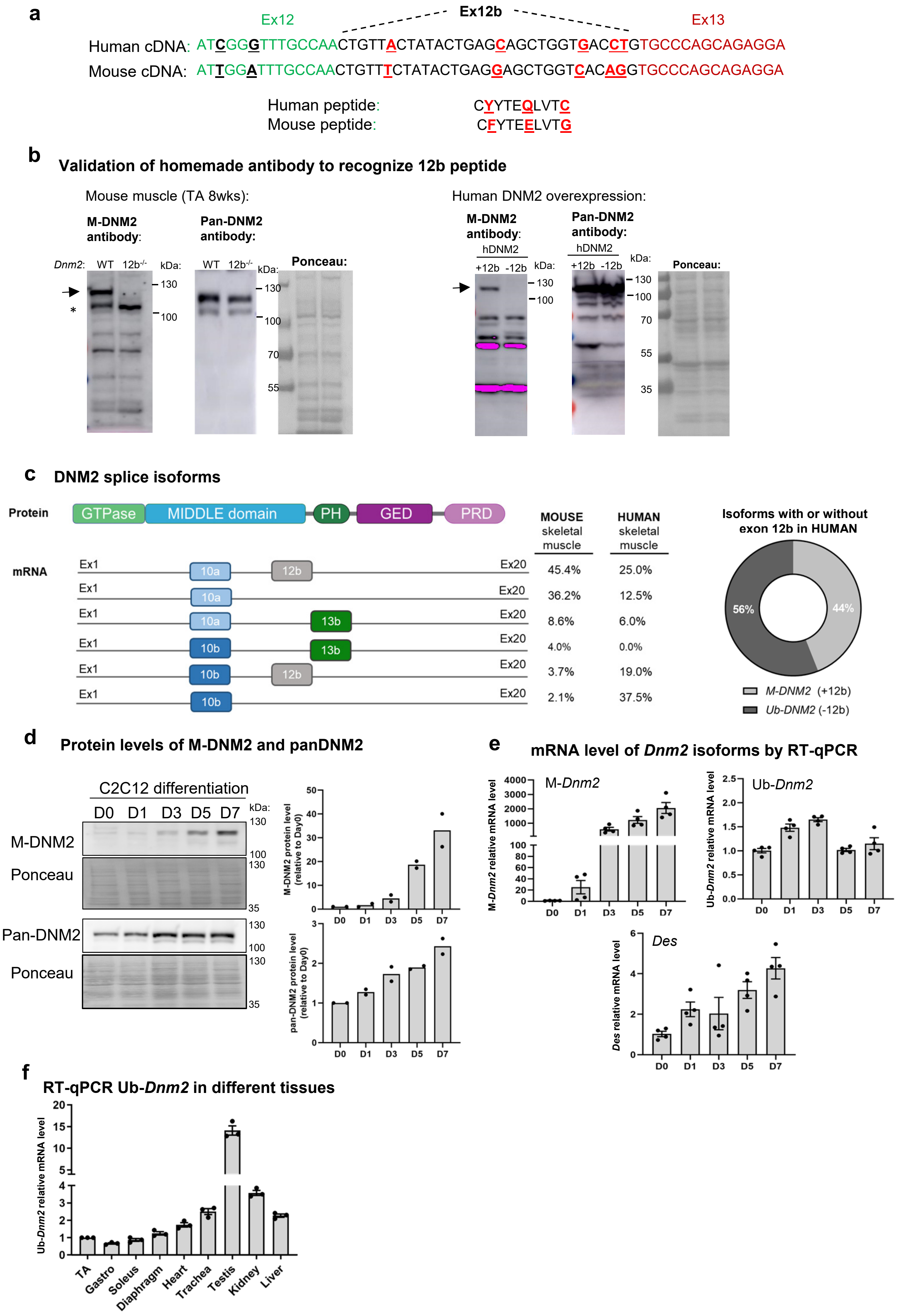

Supplementary Figure 1. M-DNM2 specific detection by novel homemade antibody and increased expression during muscle maturation (**a**) Human and mouse mRNA and peptide sequence of alternative in-frame exon 12b. Difference between mouse and human sequence are underlined. (**b**) Validation of novel homemade antibody generated to specifically detect exon 12b by western blot. Left, validation in mouse tissue using muscle protein extracts from wild-type (WT) and total knock out for exon 12b (*Dnm2*ex12b$^{-/-}$) mice. The arrow indicates the specific band for M-DNM2 and * indicates an unspecific band found in muscle protein extracts. Right, validation against human M-DNM2 using protein extract from Sf9 insect cells overexpressing human DNM2 with or without exon 12b. Specific detection against M-DNM2 was found, while both isoforms are recognized by pan-DNM2 antibody. This result was reproduced with *Dnm2*ex12b$^{-/-}$ samples as can be observed in Fig. 2b. (**c**) Left, schema of the different splice isoforms of *Dnm2* obtained by RNAseq in TA muscles of 7-week-old wild-type mice (n=4 mice) and comparison with DNM2 isoforms in human skeletal muscle obtained by RT-PCR followed by cDNA cloning and sequencing (32 clones analyzed). In the top are shown the isoforms with higher expression in mouse tissues. Isoform with exon 12b and 13b together was not found. Right, pie chart showing proportions of *DNM2* transcripts containing (M-*Dnm2*) or not (Ub-*Dnm2*) exon 12b in human skeletal muscle. (**d**) M-DNM2 and pan-DNM2 protein level in C2C12 mouse myoblast before (day0, D0) and after the start of differentiation process to myotubes (D1, D3, D5, D7). Result from 2 independent experiments with fold increase protein level compared to D0 (myoblast). (**e**) mRNA level of *Dnm2* isoforms throughout C2C12 differentiation compared to myoblast (low 12b expression) (n=4 independent experiments). *Des* (encoding desmin) is used as control of muscle differentiation. (**f**) mRNA relative expression of Ub-*Dnm2* done by RT-qPCR in several muscle and non-muscle tissues from 5-week-old wild-type mice (n=3 mice). Level of expression in different tissues was compared to expression in TA for each mouse. (**e, f**) Data are represented as mean values ± SEM. Source data are provided as a Source Data file.

Supplementary Figure 2

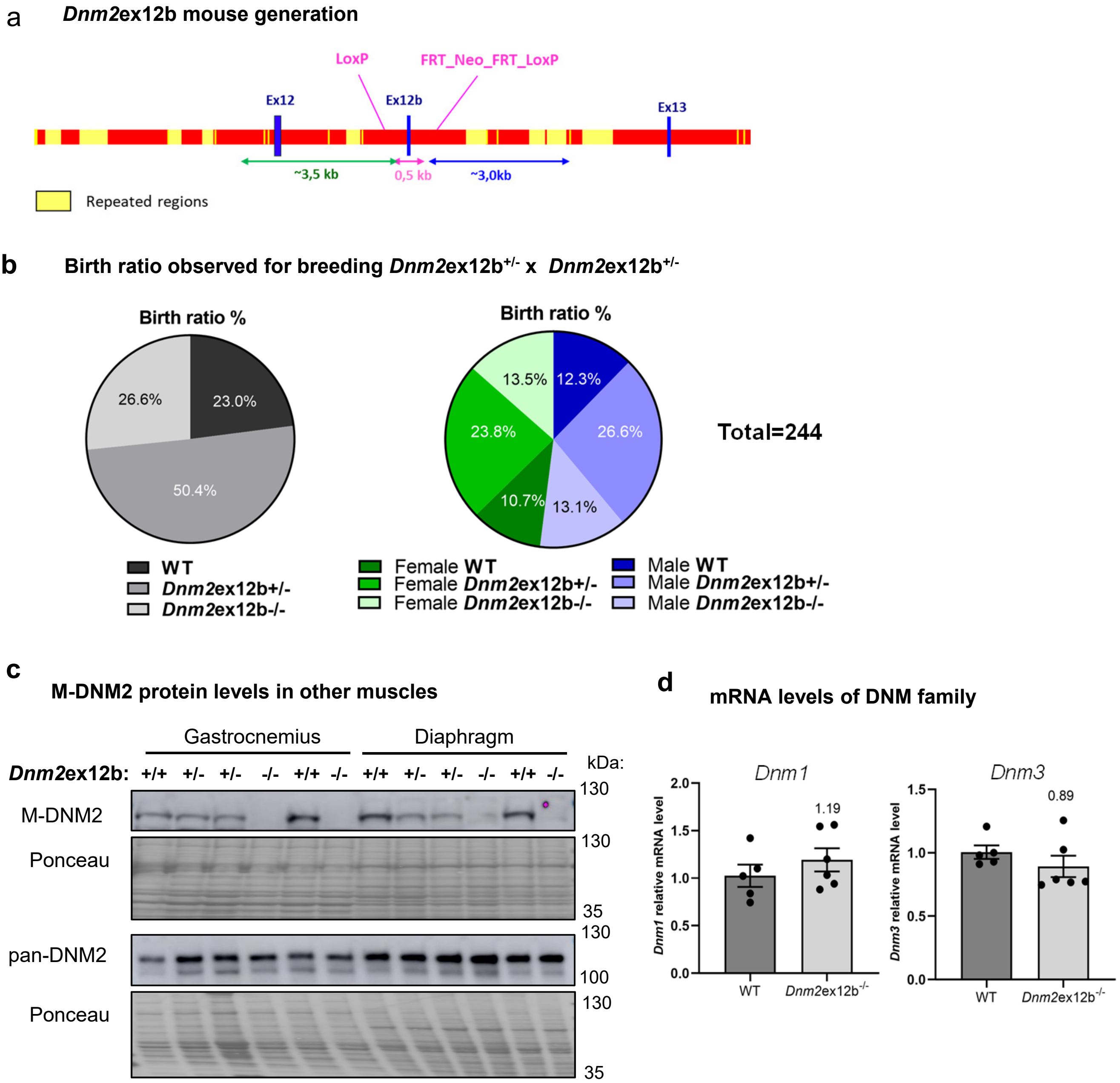

Supplementary Figure 2. Generation and validation of *Dnm2*ex12b$^{-/-}$ viable mouse model. (**a**)Targeted disruption of mouse exon 12b to create *Dnm2*ex12b$^{-/-}$ mice. It is shown the genomic region surrounding the targeted exon 12b of *Dnm2* and position of loxP sequence inserted by homologous recombination. LoxP heterozygous mice were crossed with Rosa-Cre mice leading to constative deletion of this exon. (**b**) Genotype proportion obtained 10 days after birth. No difference to expected ratio (*P*=0.9286 by two-tailed chi-square test): 25%WT, 50% *Dnm2*ex12b$^{+/-}$, 25% *Dnm2*ex12b$^{-/-}$. No difference neither taking in account males and females separately. (**c**) Western blot of gastrocnemius and diaphragm muscle lysates from WT (+/+, n=2), *Dnm2*ex12b$^{+/-}$ (+/-, n=1) and *Dnm2*ex12b$^{-/-}$ (-/-, n=2) mice probed with M-DNM2 and pan-DNM2 antibodies. These samples were run again in a technical replicate reproducing the same result. (d) mRNA levels by RT-qPCR of *Dnm1* and *Dnm3* in WT and *Dnm2*ex12b$^{-/-}$ mice. (n=5 mice, not significant by two-tailed unpaired t test). Data is represented as mean values ± SEM. Source data is provided as a Source Data file.

Supplementary Figure 3

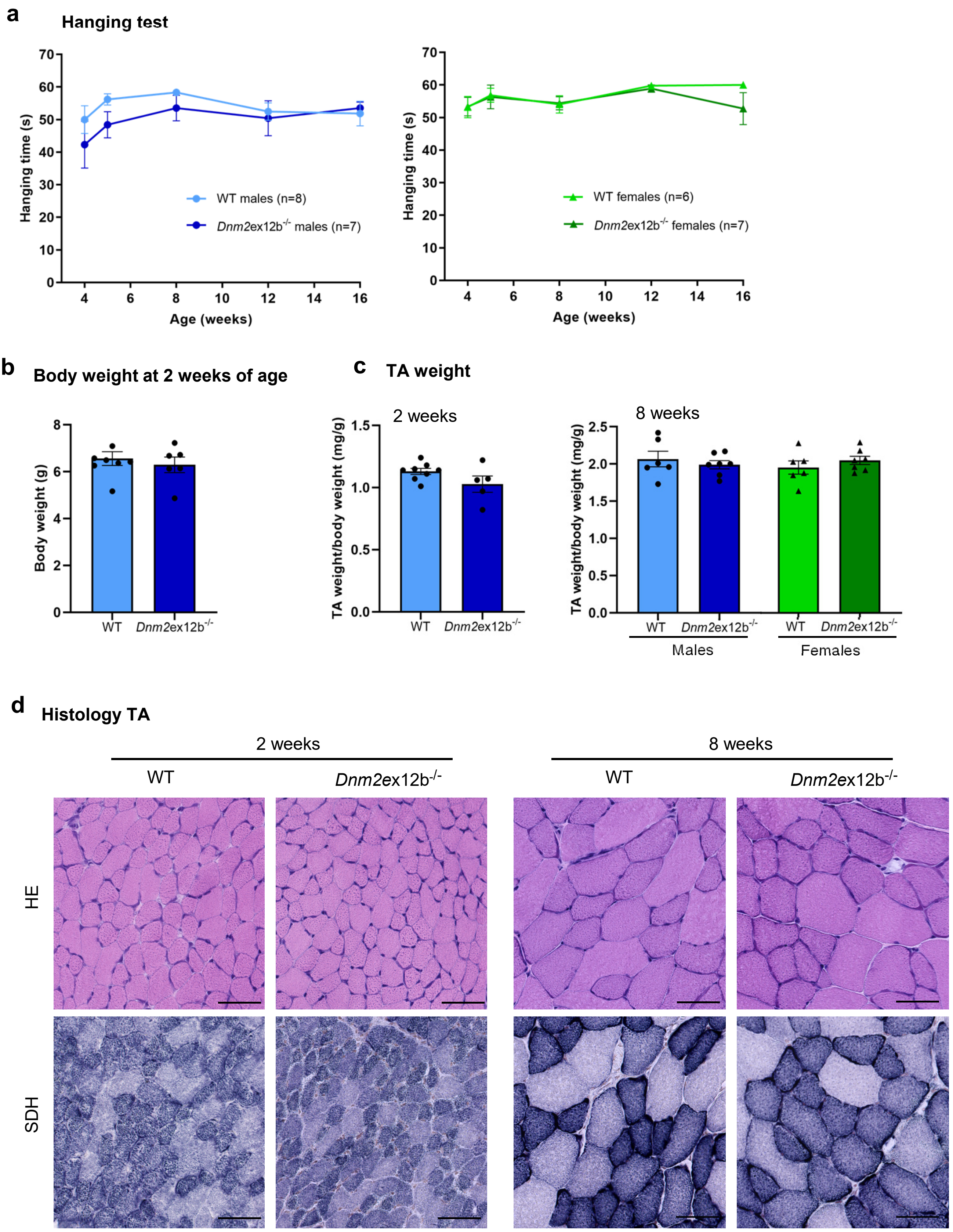

Supplementary Figure 3. Deletion of exon 12b does not cause any muscle phenotype at 2 weeks of age. (**a**) Hanging performance of WT and *Dnm2*ex12b$^{-/-}$ mice at different time points. Curves show averages values ±SEM for each time point and genotype. (**b**) Body weight of 2-week-old male WT (n=8) and *Dnm2*ex12b$^{-/-}$ (n=6) mice/group (not significant (ns) by two-tailed unpaired t test). (**c**) TA muscle weight ratio calculated at 2 and 8wks in WT (2wks, n=8; 8wks, n=6) and *Dnm2*ex12b$^{-/-}$ (2wks, n=6; 8wks, n=7) male mice (ns by two-tailed unpaired t test). (**d**) Representative image of H&E and SDH staining of TA muscle section from WT and *Dnm2*ex12b$^{-/-}$ male mice at 2 and 8wks of age. Scale bar =50 µm. Muscle sections for at least three mice for each condition were qualitatively compared and analyzed for abnormalities at histological level. (**b, c**) Data are represented as mean values ± SEM. Source data are provided as a Source Data file.

Supplementary Figure 4

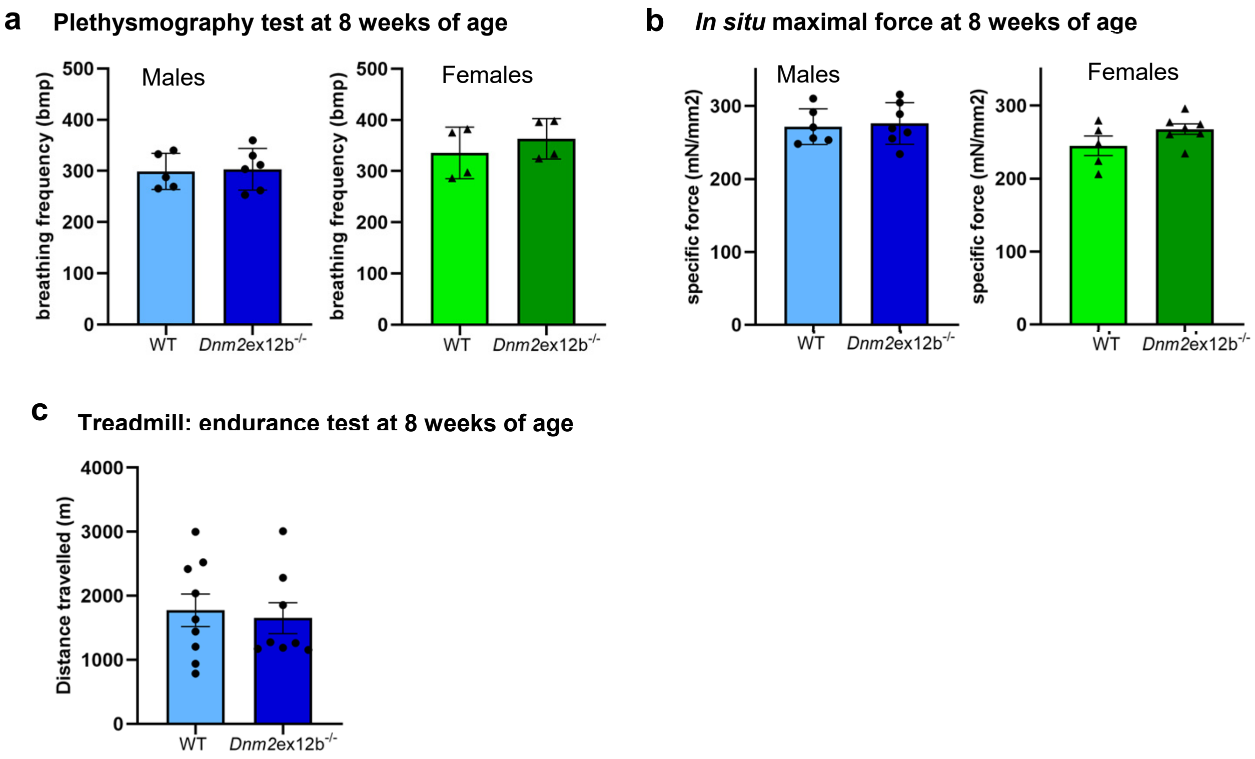

Supplementary Figure 4. Deletion of exon 12b does not affect muscle performance and histology at 8 weeks of age. (**a**) Plethysmograph result of breathing frequency parameter, unit bmp (breathing frequency parameter). The plethysmograph test was performed on resting mice to assess resting breathing patterns (WT males (n=5) and *Dnm2*ex12b$^{-/-}$ (n=6), females WT (n=4) and *Dnm2*ex12b$^{-/-}$ (n=4) mice/group; not significant (ns) by two-tailed unpaired t test). (**b**) Specific maximal force of the TA measured *in-situ* from WT (male, n=6; female, n=5) and *Dnm2*ex12b$^{-/-}$ (male and female, n=7) mice (ns by two-tailed unpaired t test). (**c**) Distance travelled until exhaustion during treadmill test done in 8-week-old WT (n=9) and *Dnm2*ex12b$^{-/-}$ (n=8) male mice (ns by two-tailed Mann-Whitney test). (**a-c**) Data are represented as mean values ± SEM. Source data are provided as a Source Data file.

Supplementary Figure 5

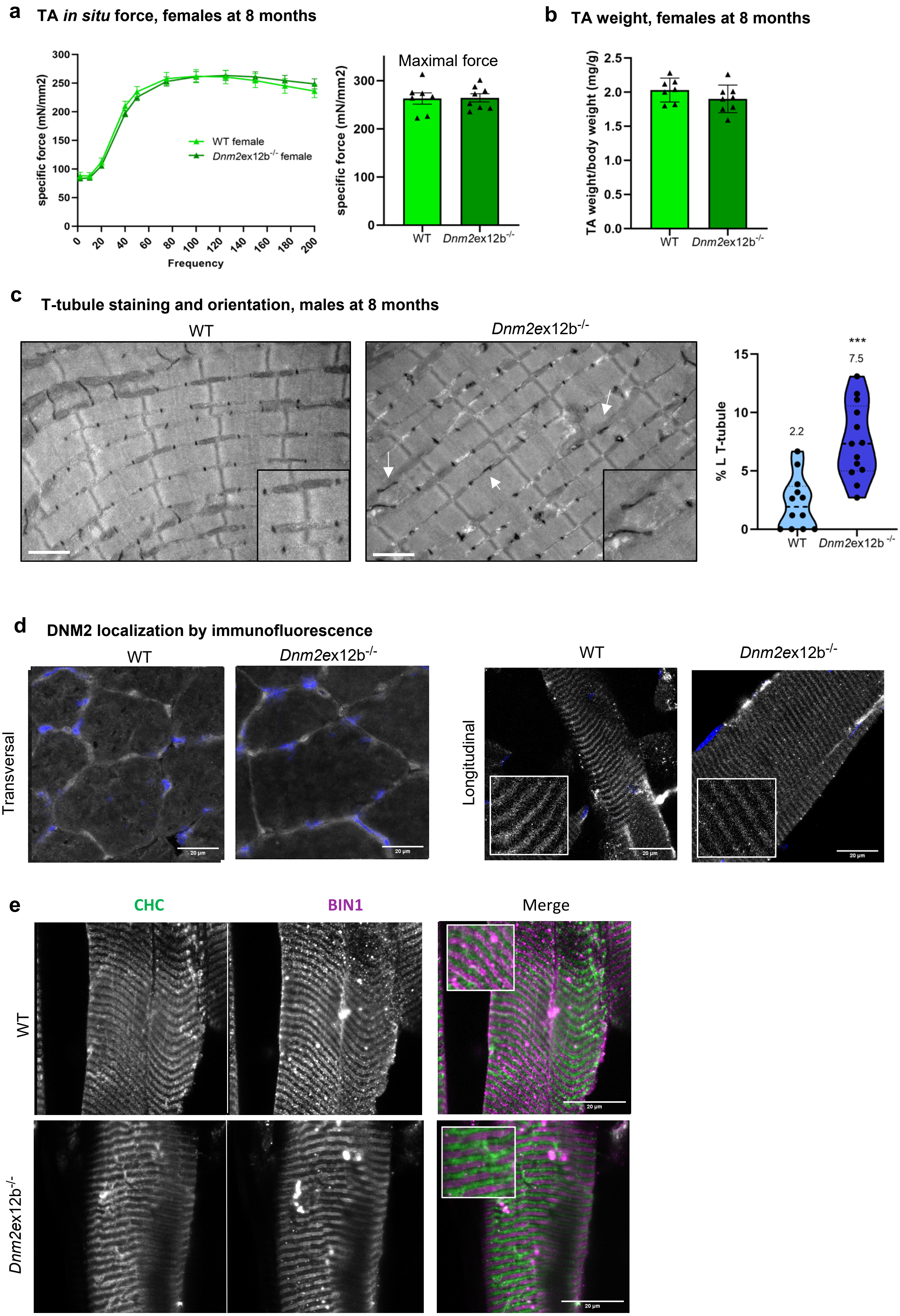

Supplementary Figure 5. Deletion of exon 12b does not affect female muscle performance at 8 months neither location of DNM2 and partners but cause slight mis-orientation of T-tubules. (a) Specific muscle force produced by TA of WT (n=7) and *Dnm2*ex12b$^{-/-}$ (n=8) 8-month-old female mice when stimulated at different frequencies. Right, specific maximal force is represented (ns (not significant) by two-tailed unpaired t test). (**b**) TA weight ratio with body weight of WT (n=7) and *Dnm2*ex12b$^{-/-}$ (n=8) female mice at 8 months of age (ns by two-tailed unpaired t test). (**c**) Electron microscopy images from TA muscle of WT and *Dnm2*ex12b$^{-/-}$ 8-month-old male mice labeled with potassium ferrocyanide staining. Scale bar=1µm. Higher magnification is shown for each condition. Arrows indicate T-tubules with longitudinal orientation (L T-tubules). This misoriented T-tubules were quantified for at least 5 different fibers in 2 mice per group, n= 12 fiber images analyzed for WT genotype and n=13 for *Dnm2*ex12b$^{-/-}$ genotype (***P= 0.0001 by two-tailed unpaired t test). (**d**) Representative images of DNM2 localization by immunofluorescence observed in transversal and longitudinal sections of WT and *Dnm2*ex12b$^{-/-}$. Scale bar=20µm. (**e**) Longitudinal TA muscle sections stained with BIN1 and CHC (Clathrin Heavy Chain) antibodies. Scale bar=20µm. (**a, b**) Data are represented as mean values ± SEM. Source data are provided as a Source Data file. (**d, e**) Staining result was reproduced in a biological replicate using a muscle section from a different mouse in an independent experiment.

Supplementary Figure 6

a **Observed outcome of females for breeding Mtm1$^{+/-}$ *Dnm2*ex12b$^{+/-}$ x *Dnm2*ex12b$^{+/-}$**

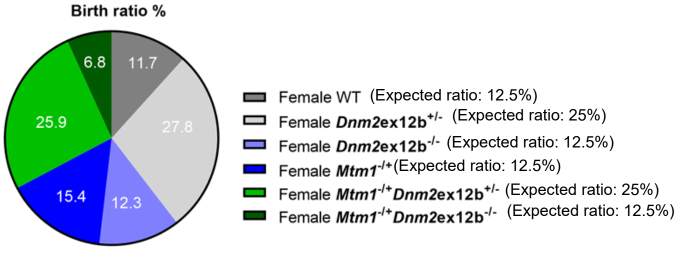

b **Observed outcome of males and females for breeding Mtm1$^{+/-}$ x *Dnm2*ex12b$^{+/-}$**

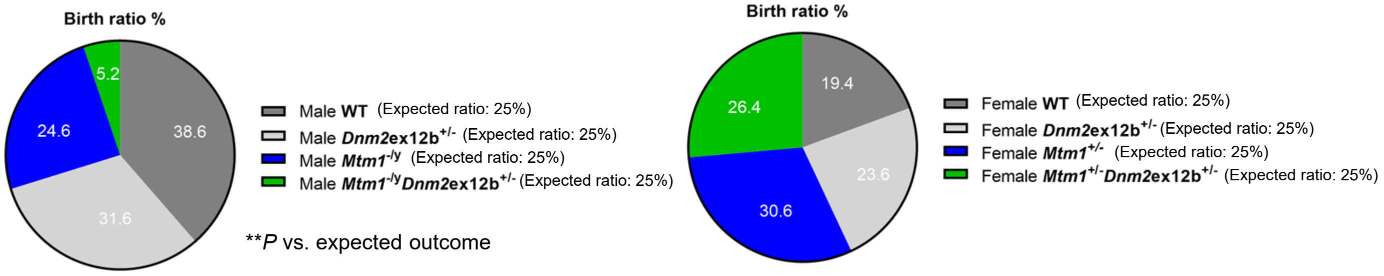

c **Muscle weights males 4 weeks old**

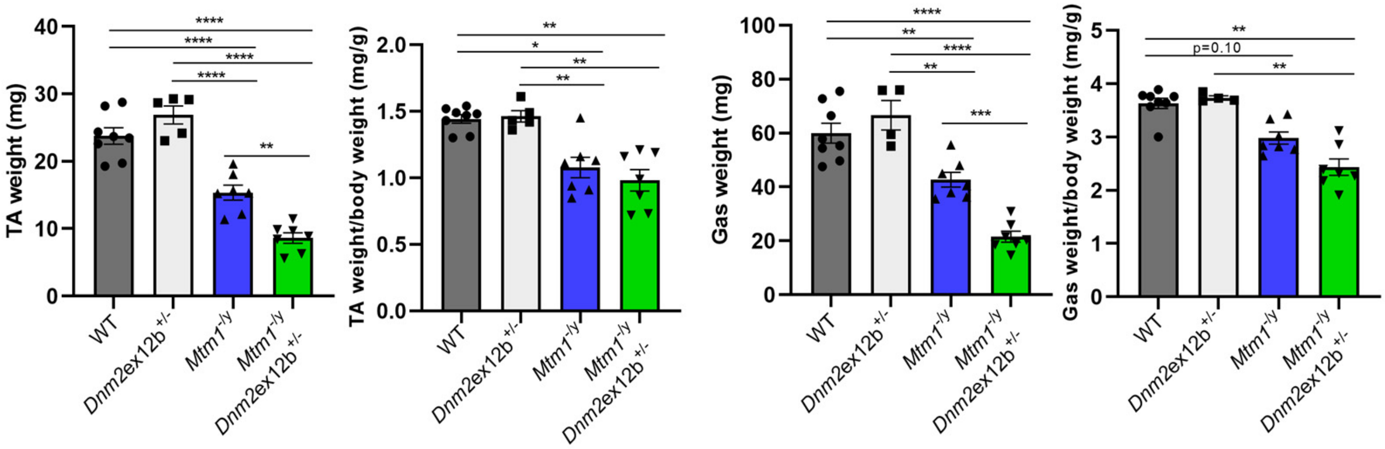

d **Fiber size average**

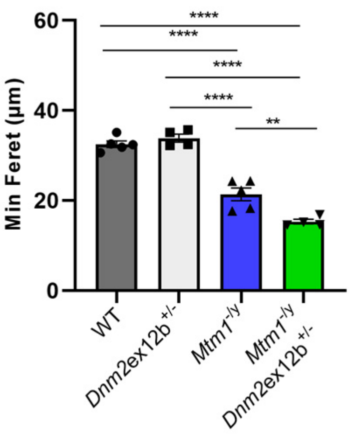

e **Large fibers**

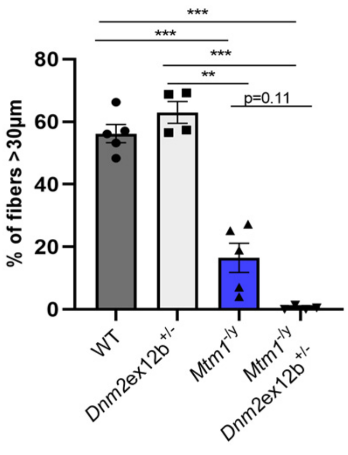

Supplementary Figure 6. *Dnm2* splice switching towards ubiquitous isoforms in XLCNM has an impact in viability of mice and increase hypotrophy of muscle fibers. (**a**) Genotype proportion obtained 10 days after birth for females. No difference to expected ratio (P=0.3298, two-tailed chi-square test). (**b**) Genotype proportion obtained 10 days after birth for females and males. Difference to Mendelian ratio was obtained for males (**P=0.0073, two-tailed chi-square test). No difference to expected ratio was obtained for females (P=0.5958, two-tailed chi-square test). (**c**) Muscle weights for TA (left) and gastrocnemius (right) of WT (n=8), *Dnm2*ex12b$^{-/-}$ (n=5), *Mtm1*$^{-/y}$ (n=7) and *Mtm1*$^{-/y}$ *Dnm2*ex12b$^{-/-}$ (n=7) at 4 weeks of age. TA weight, **P= 0.0014 and Gas weight from top to bottom **P= 0.0041, **P= 0.0011, ***P= 0.0007 by one-way ANOVA with Tukey's post hoc test. TA weight/ body weight, from top to bottom: **P= 0.0038, *P=0.0126, **P= 0.0028, **P= 0.0098 by Brown-Forsythe and Welch ANOVA with Dunnett's T3 post hoc test. Gastro weight/ body weight, from top to bottom: **P= 0.0020, **P=0.0058 by Kruskal-Wallis test with Dunn's post hoc test. For all ****P<0.0001. (**d**) Fiber size average for TA muscle section of 4-week-old male WT (n=5), *Dnm2*ex12b$^{+/-}$ (n=4), *Mtm1*$^{-/y}$ (n=5), *Mtm1*$^{-/y}$ *Dnm2*ex12b$^{+/-}$ (n=4) mice (**P=0.0011, ****P<0.0001 by Brown-Forsythe and Welch ANOVA with Dunnett's T3 post hoc test). (**e**) Percentage of fibers higher than 30 µm of mice in (d) (from top to bottom ***P= 0.001, ***P= 0.0009, ***P= 0.0005, **P= 0.0011 by Brown-Forsythe and Welch ANOVA with Dunnett's T3 post hoc test). (**c-e**) Data are represented as mean values ± SEM. Source data are provided as a Source Data file.

Supplementary Figure 7

a **Histology of diaphragm at 4 weeks old**

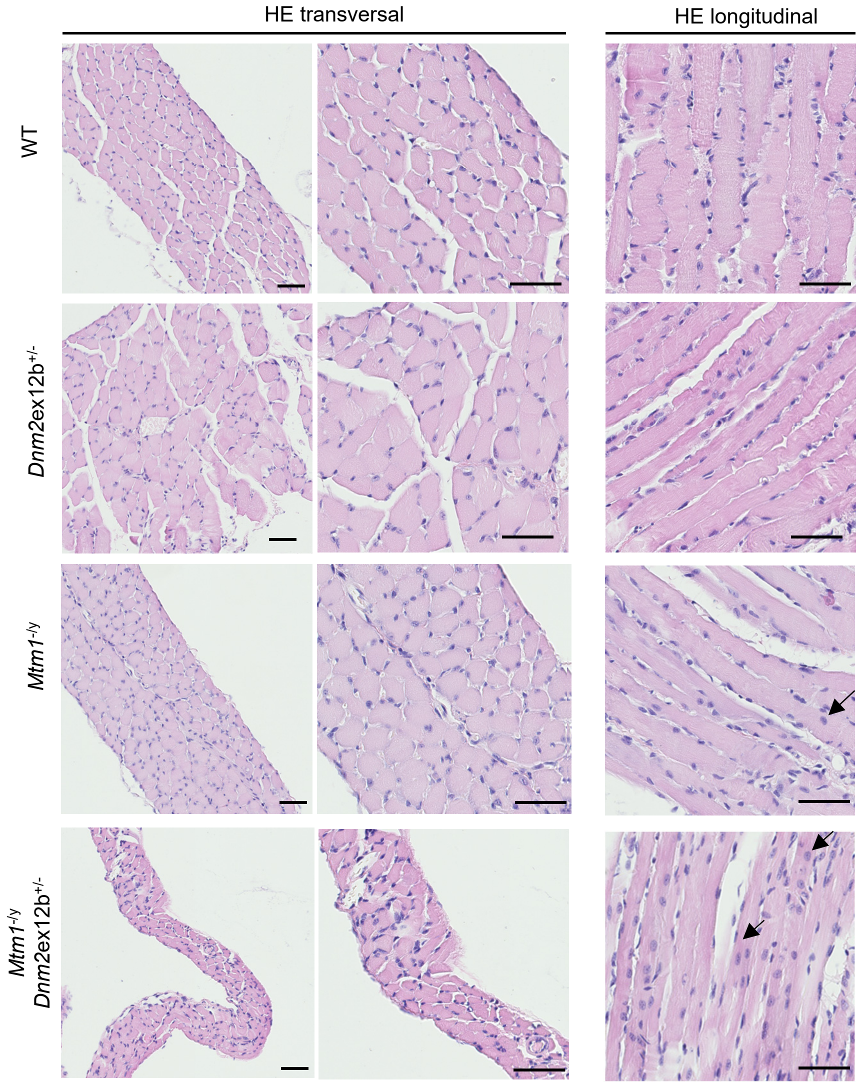

b **Diaphragm weight**

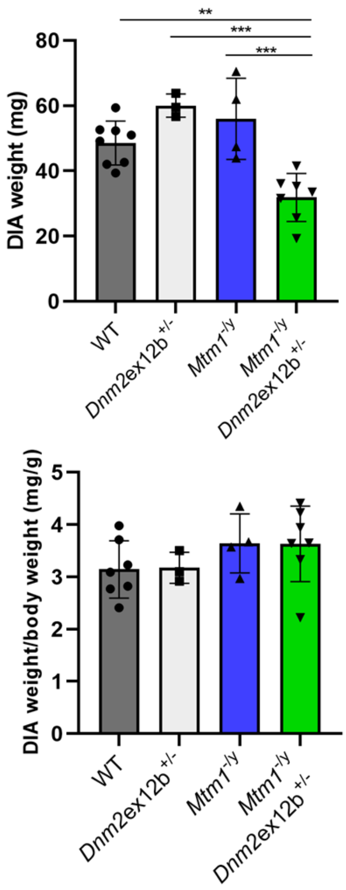

c **Ultrastructure TA at 4 weeks old**

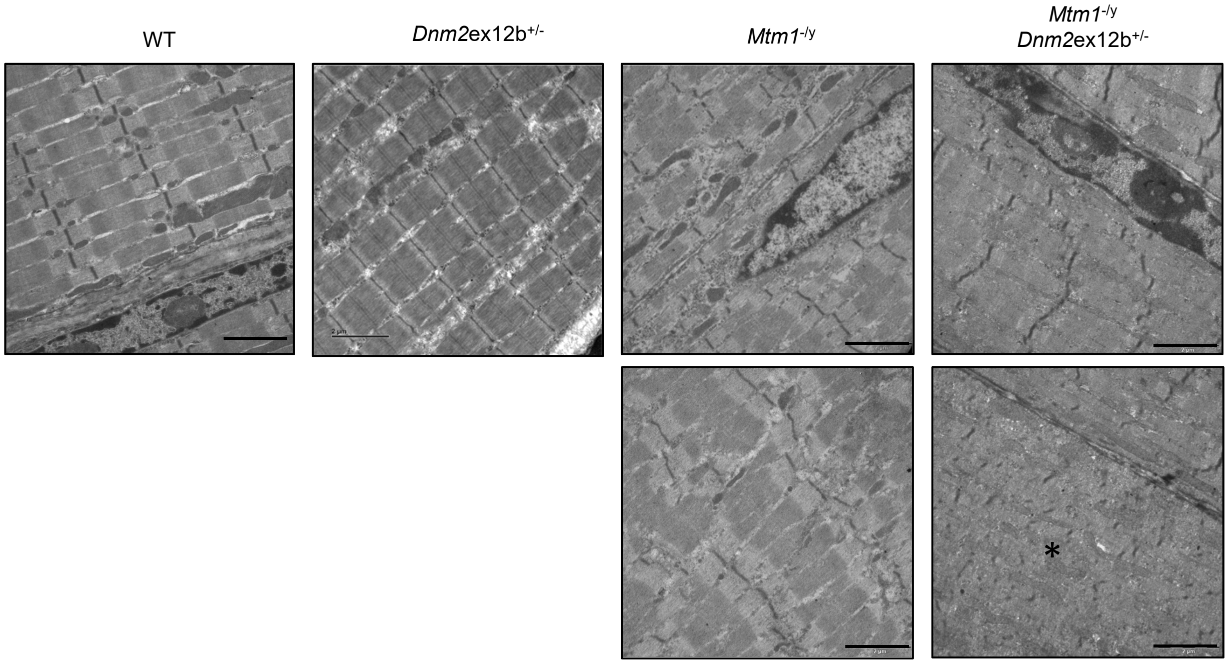

Supplementary Figure 7. *Dnm2* splice switching in XLCNM worsens diaphragm histology and results in highly disorganized sarcomere structure in skeletal muscle. (**a**) Representative image of diaphragm histology of mice at 4 weeks of age. Scale bar=50 µm. Arrows indicate fibers with internalize nuclei. (**b**) Diaphragm weight and ratio with body weight of WT (n=8), *Dnm2*ex12b$^{+/-}$ (n=3), *Mtm1*$^{-/y}$ (n=4), *Mtm1*$^{-/y}$ *Dnm2*ex12b$^{+/-}$ (n=7) 4-week-old mice (**P=0.0036, ***P=0.0004, ***P=0.0007 by one-way ANOVA with Tukey's post hoc test). Data are represented as mean values ± SEM. Source data are provided as a Source Data file. (**c**) Representative images of electron microscopy from TA of 4-week-old mice. Similar results were obtained in a biological replicate at same age . Asterisk (*) indicates a fiber with totally disrupted Z-line and sarcomere structure. Scale bar=2µm.

Supplementary Figure 8

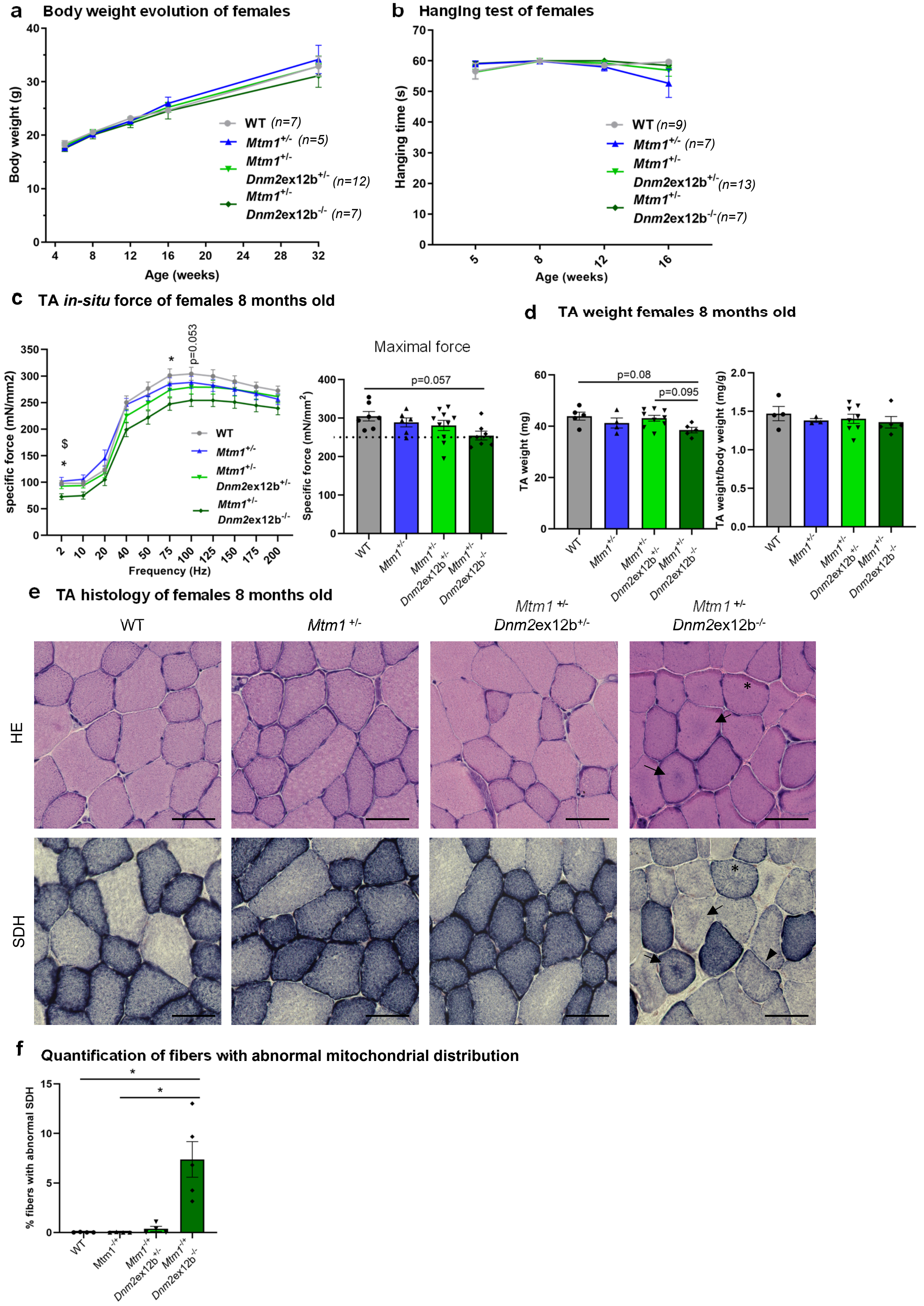

Supplementary Figure 8. *Dnm2* splice switching in Mtm1$^{+/-}$ females cause the apparition of a mild muscle phenotype with CNM hallmarks in histology. (**a**) Evolution of body weight of females from 5 weeks to 8 months of age (‘n’ mice/ group indicated in the figure). (**b**) Hanging test performance during maximum time of 60 seconds from 5 weeks to 4 months (‘n’ mice/group indicated in the figure). (**c**) Specific muscle force produced by TA muscle of 8-month-old female WT (n=7), *Mtm1*$^{+/-}$ (n=6), *Mtm1*$^{+/-}$ *Dnm2*ex12b$^{+/-}$ (n=10), *Mtm1*$^{+/-}$ *Dnm2*ex12b$^{-/-}$ (n=7) mice when stimulated at different frequencies. Right, specific maximal force is represented (comparison at each frequency *Mtm1*$^{+/-}$ *Dnm2*ex12b$^{-/-}$ vs. WT * and vs. *Mtm1*$^{+/-}$ \$: *P=0.0226, \$$P$=0.0385 at 2Hz and *$P$=0.0375 at 75Hz by one-way ANOVA with Tukey’s post hoc test). (**d**) TA muscle weight and ratio with body weight (WT (n=5), *Mtm1*$^{+/-}$ (n=4), *Mtm1*$^{+/-}$ *Dnm2*ex12b$^{+/-}$ (n=9), *Mtm1*$^{+/-}$ *Dnm2*ex12b$^{-/-}$ (n=5) mice/group; statistical analysis by one-way ANOVA with Tukey’s post hoc test). (**e**) Representative HE and SDH staining of TA transversal sections from 8-month-old female mice. Arrows and arrowhead indicate fibers with central or peripheral accumulations of oxidative staining, respectively. Asterik (*) shows a fiber with ‘spoke of wheels’ pattern. Scale bar =50 µm. (**f**) Percentage of fibers with abnormal SDH staining as the fibers indicated in the previous figure (WT, *Mtm1*$^{+/-}$, and *Mtm1*$^{+/-}$ *Dnm2*ex12b$^{+/-}$ (n=4), *Mtm1*$^{+/-}$ *Dnm2*ex12b$^{+/-}$ (n=5) mice/group; >1000 fibers/mouse analyzed; from top to bottom *P=0.0209, *P= 0.0267 by Kruskal-Wallis test with Dunn’s post hoc test). (**a-d, f**) Data are represented as mean values ± SEM. Source data are provided as a Source Data file.

Supplementary Figure 9

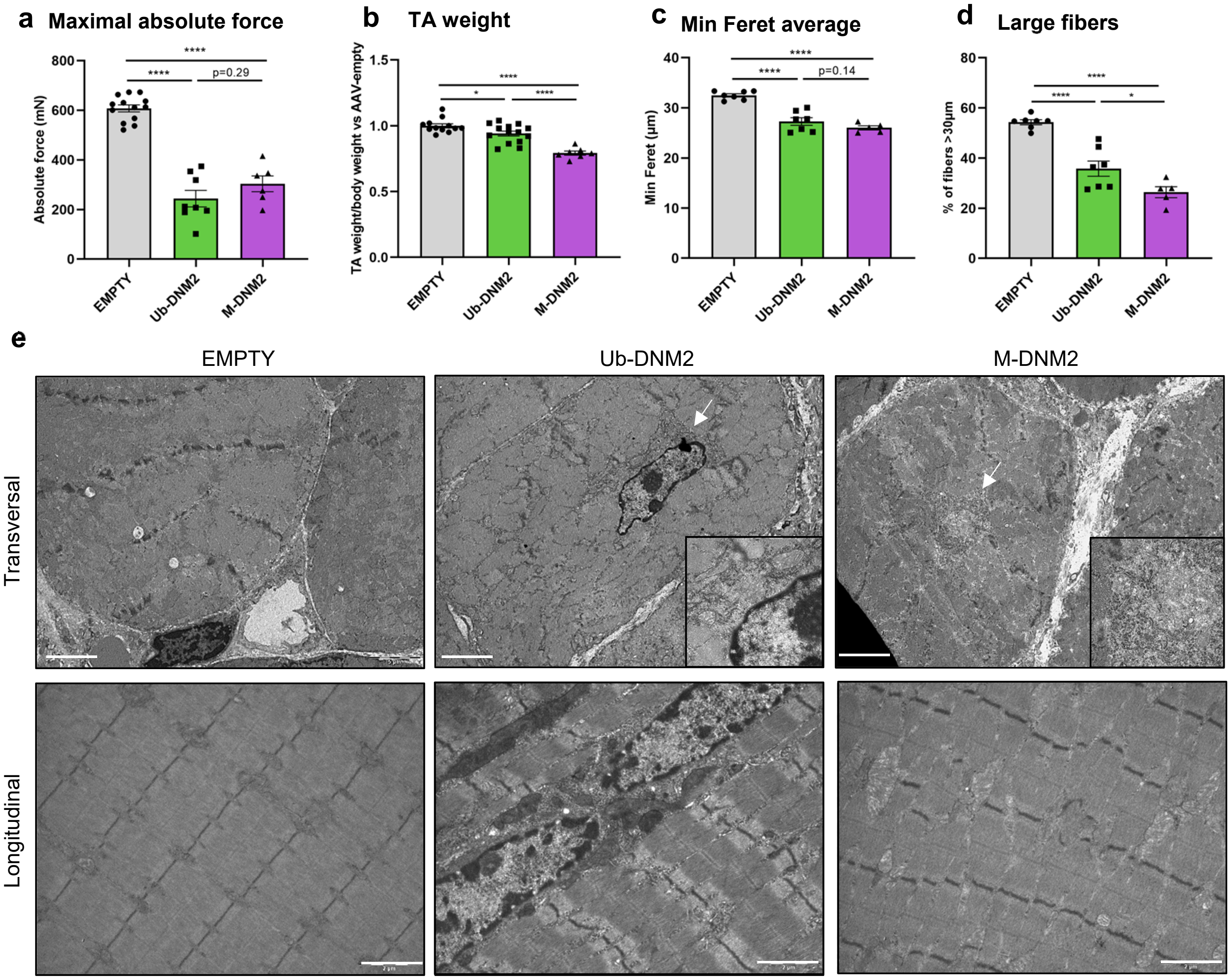

Supplementary Figure 9. Overexpression of Ub-DNM2 and M-DNM2 correlates with muscle hypotrophy and shows different CNM hallmarks in muscle ultrastructure. (**a**) Absolute maximal force measured 2 weeks after injection (Empty (n=13), Ub-DNM2 (n=8), M-DNM2 (n=6) mice/group; *****P*<0.0001 by one-way ANOVA with Tukey's post hoc test) (**b**) Ratio of TA weight and total body weight compared to muscles injected with empty virus 2 weeks after injection (Empty (n=12), Ub-DNM2 (n=14), M-DNM2 (n=7) mice/group, *****P*<0.0001, **P*=0.0442 by one-way ANOVA with Tukey's post hoc test) (**c**) Average of minimum Feret of TA fibers (Empty (n=7), Ub-DNM2 (n=7), M-DNM2 (n=5) mice/group, *****P*<0.0001 by one-way ANOVA with Tukey's post hoc test). (**d**) Percentage of fibers higher than 30 µm of same mice as (c) (*****P*<0.0001,**P*=0.0329 by Kruskal-Wallis test with Dunn's post hoc test). (**e**) Representative images of electron microscopy from transversal and longitudinal sections of TA muscle. Arrows indicate the area amplified showed in low right corner. Scale bar=5µm above, scale bar=2µm below**.** Similar results were obtained in a biological replicate at the same age . (**a-d**) Data are represented as mean values ± SEM. Source data are provided as a Source Data file

Supplementary Figure 10

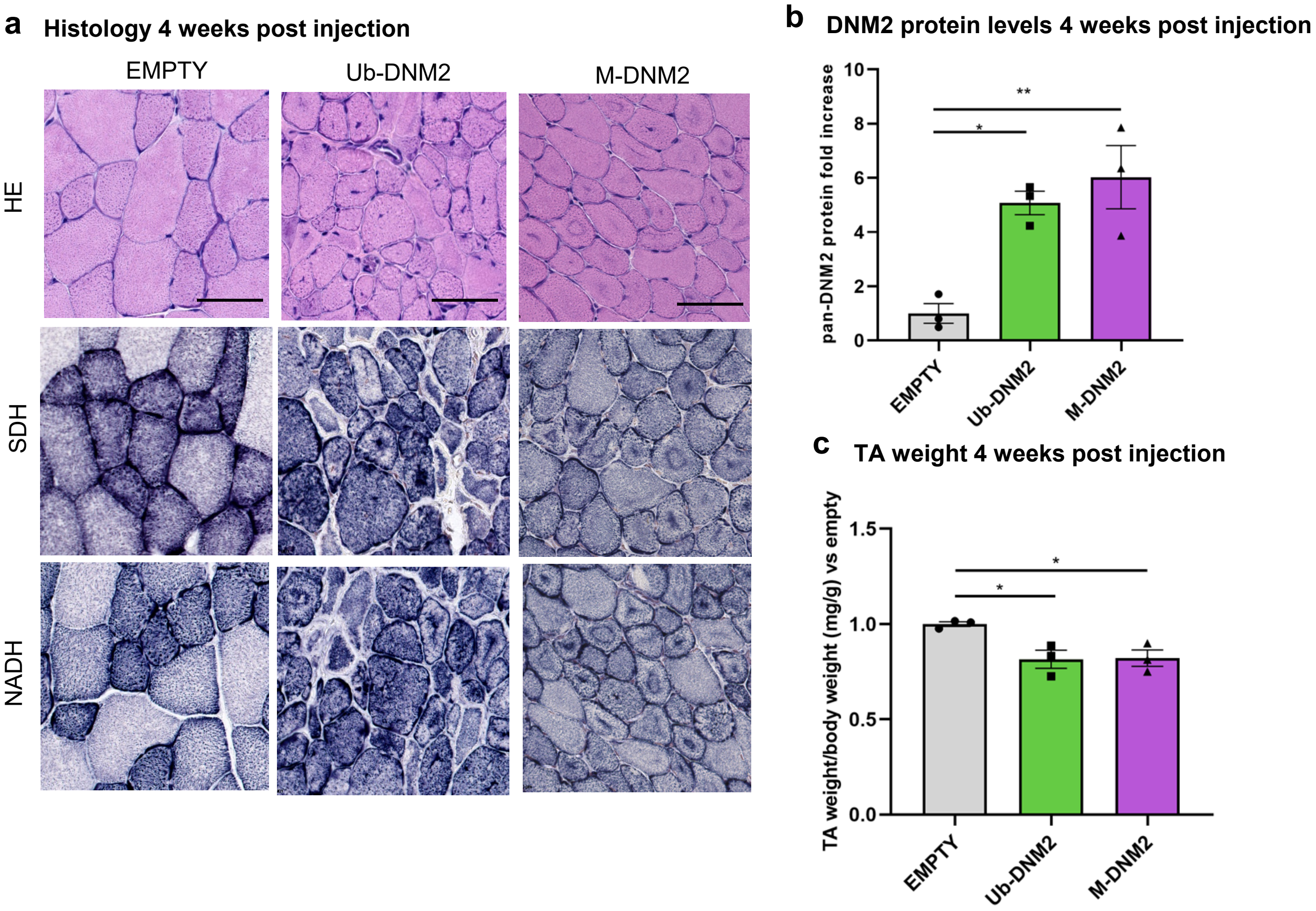

Supplementary Figure 10. Overexpression of Ub-DNM2 and M-DNM2 correlate with different CNM phenotypes also 4 weeks post injection. (**a**) Representative HE, SDH and NADH staining of TA transversal sections from mice 4 weeks after AAV injection. Scale bar =50 µm. (**b**) pan-DNM2 protein levels compared to muscle injected with AAV-Empty virus 4 weeks after injection (n=3 mice/group; **P*=0.0197, ***P*=0.0075 by one-way ANOVA with Tukey's post hoc test). (**c**) Ratio of TA weight and total body weight compared to muscles injected with AAV-Empty virus 4 weeks after injection (n=3 mice/group; Empty vs. Ub-DNM2 **P*=0.0303, and vs. M-DNM2 **P*=0.0344 by one-way ANOVA with Tukey's post hoc test). (**b-c**) Data are represented as mean values ± SEM. Source data are provided as a Source Data file.

Supplementary Figure 11

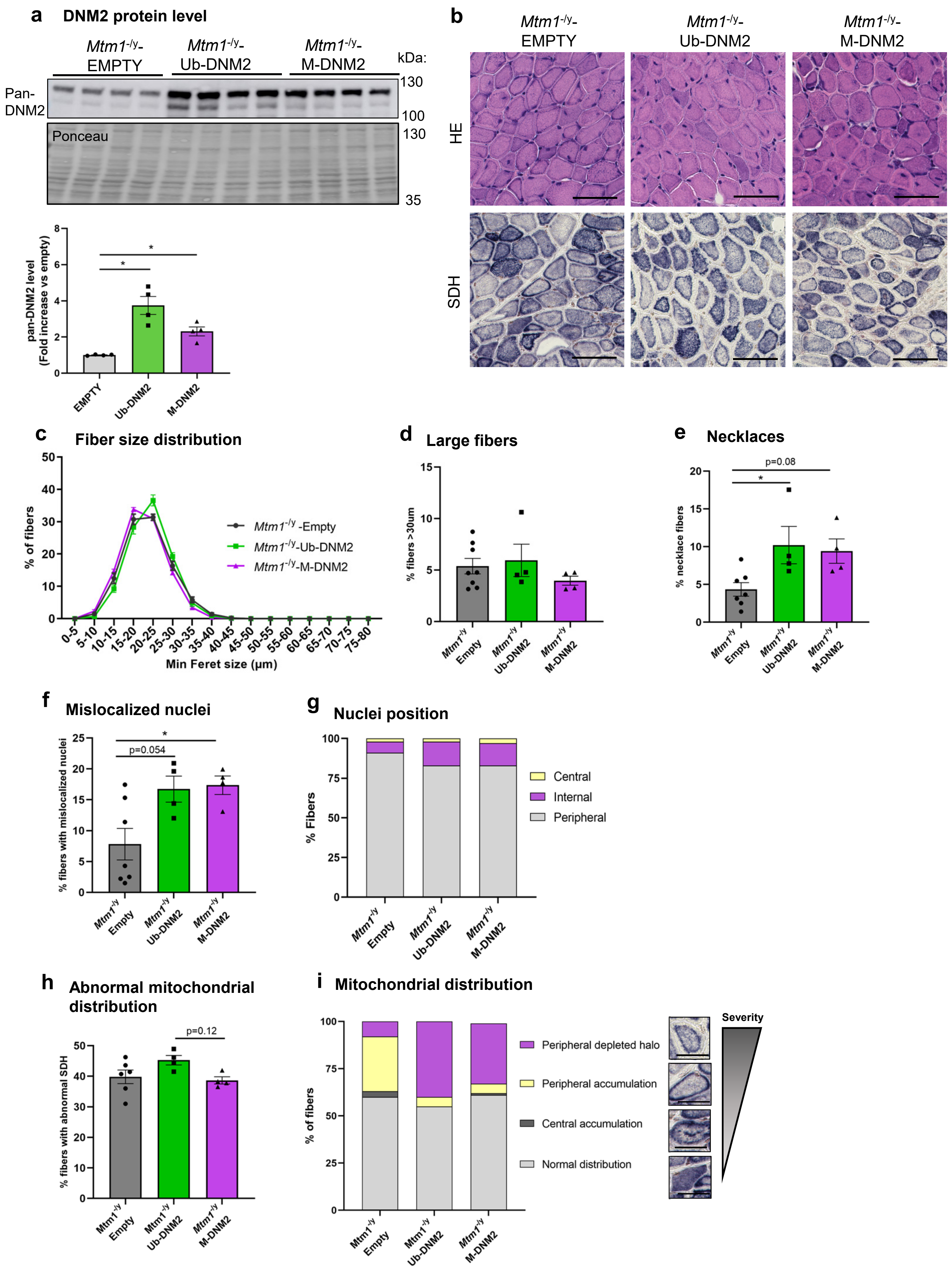

Supplementary Figure 11. Overexpression of Ub-DNM2 and M-DNM2 leads to slight aggravation of *Mtm1*$^{-/y}$ muscle phenotypes 2 weeks post-injection. (a) pan-DNM2 protein levels compared to *Mtm1*$^{-/y}$ muscle injected with AAV-Empty virus in 4 mice/group (Empty vs. Ub-DNM2 *P= 0.0237 and vs. M-DNM2 *P= 0.0256, not significant (ns) Ub-DNM2 vs. M-DNM2 by Brown-Forsythe and Welch ANOVA with Dunnett's T3 post hoc test). (**b**) Representative HE, SDH staining of TA transversal sections from *Mtm1*$^{-/y}$ mice. Scale bar =50 µm. Results reproduced and histological parameters quantified for more mice as shown in next figures. **(c)** Minimum Feret diameter (MinFeret) of TA fibers grouped into 5 µm intervals, including fibers with size bigger than lower limit and smaller or equal than upper limit (*Mtm1*$^{-/y}$ Empty (n=8), *Mtm1*$^{-/y}$ Ub-DNM2, M-DNM2 (n=4) mice/group). (**d**) Percentage of fibers in (c) higher than 30 µm (ns by Kruskal-Wallis test with Dunn's post hoc test) (**e**) Percentage of fibers with necklaces (*Mtm1*$^{-/y}$ Empty (n=7), *Mtm1*$^{-/y}$ Ub-DNM2, M-DNM2 (n=4) mice/group; *P= 0.043 by one-way ANOVA with Tukey's post hoc test). (**f**) Percentage of fibers with mislocalized nuclei (centralized or internalized) (*Mtm1*$^{-/y}$ Empty (n=7), *Mtm1*$^{-/y}$ Ub-DNM2, M-DNM2 (n=4) mice/group; *P= 0.0393 by one-way ANOVA with Tukey's post hoc test). (**g**) Fibers of (f) classified by nuclei position. (**h**) Percentage of fibers with abnormal SDH staining (*Mtm1*$^{-/y}$ Empty (n=7), *Mtm1*$^{-/y}$ Ub-DNM2, M-DNM2 (n=6) mice/group one-way ANOVA with Tukey's post hoc test). (**i**) Fibers of (h) classified by mitochondrial distribution analyzed using SDH staining in 4 main groups: normal distribution, with central or peripheral accumulation of SDH activity in the myofiber or with a depleted halo of SDH activity in the periphery of the fibers. Representative myofiber of each situation is shown. The pattern with pale peripheral halo corresponds to the highest severity in the muscle disease progression. For all the histological analysis more than 1000 fiber per mouse were quantified. Scale bar =25 µm. (**a, c-f, h**) Data are represented as mean values ± SEM. Source data are provided as a Source Data file. (**g, i**) Average proportion of the group is shown (individual values in Source Data File).

Supplementary Figure 12

a **DHPR localization in AAV-transduced muscles**

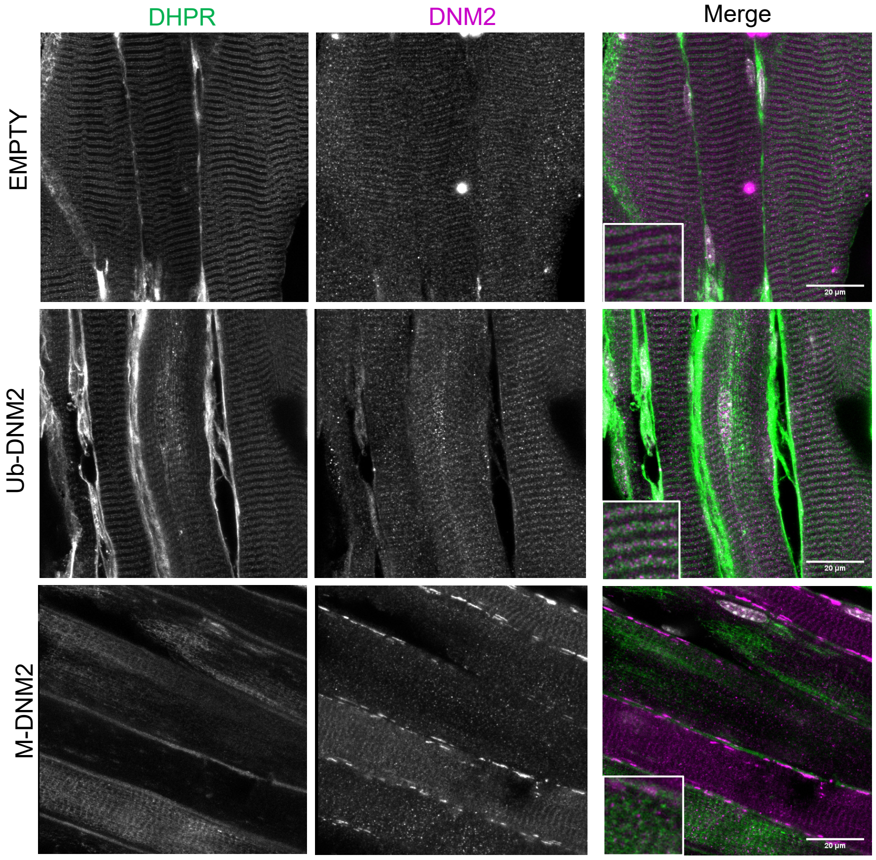

b **T-tubule staining in AAV-transduced muscles**

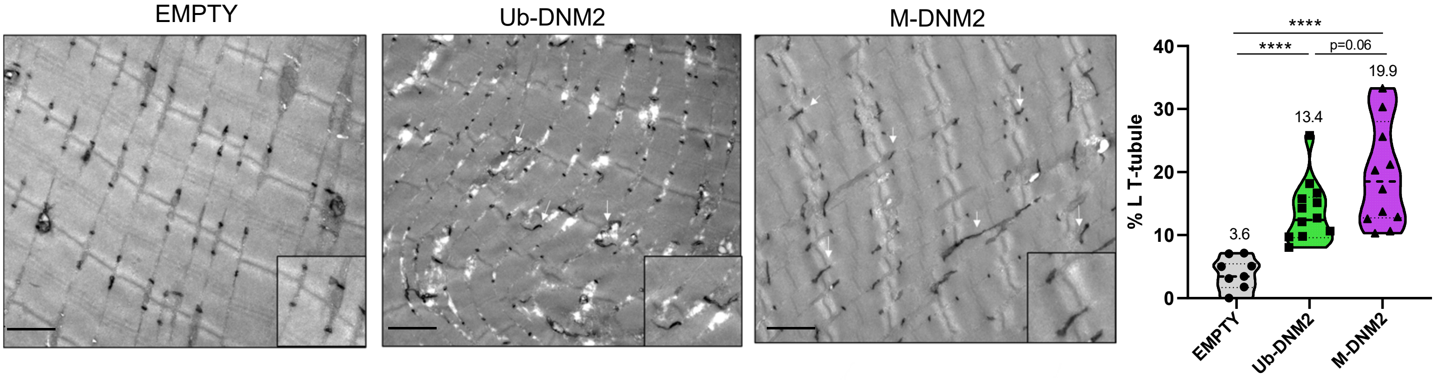

c **CHC and DNM2 colocalization, vesicular fixation**

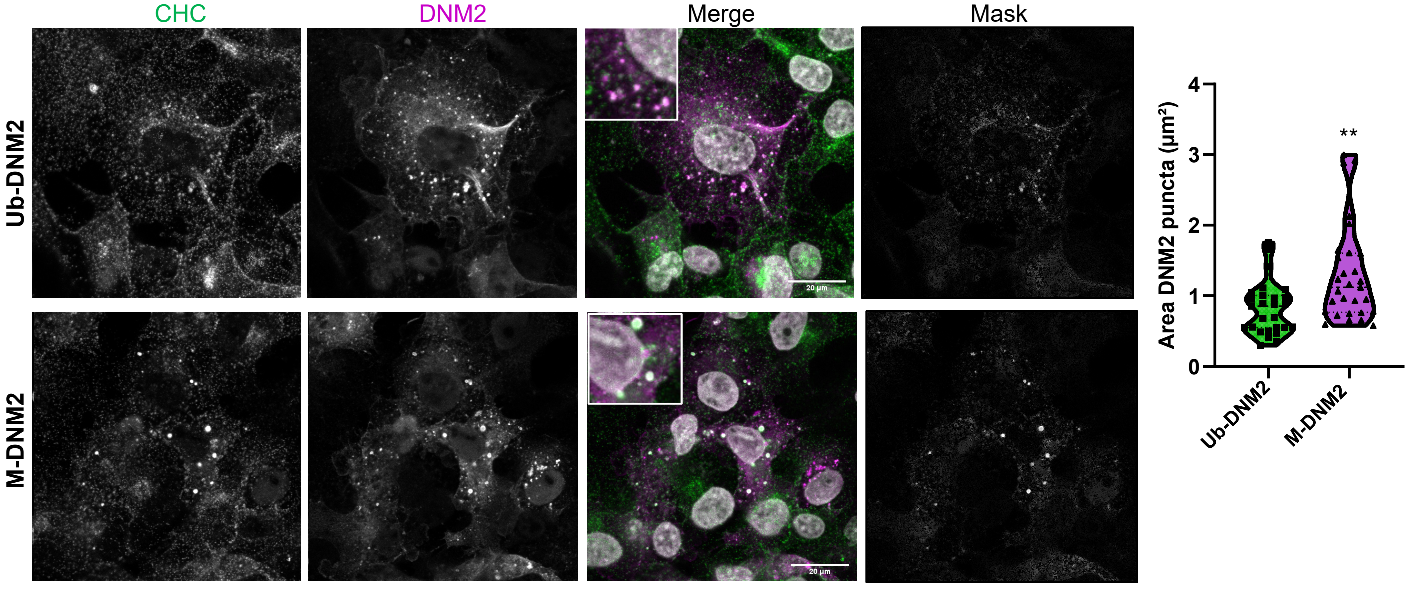

Supplementary Figure 12. M-DNM2 overexpression highly impact T-tubule organization and shows enlarge vesicular pattern in cells. (**a**) Longitudinal sections from AAV-transduced muscles stained with DHPR and DNM2 antibodies. Scale bar=20 µm. Staining result was reproduced in a biological replicate using a muscle section from a different mouse in an independent experiment. **(b**) Representative electron microscopy images of AAV-transduced muscles with T-tubule labelling. Scale bar=1µm. Higher magnification is shown for each condition. Arrows indicate T-tubules with longitudinal orientation (L-tubules). This result was reproduced and quantified in different images collected from at least two different mouse samples and the overall quantification is represented at the right (*****P*<0.0001 by Brown-Forsythe and Welch ANOVA with Dunnett's T3 post hoc test). (**c**) Colocalization of CHC and exogenous DNM2 by immunofluorescence in COS-1 cells with enrichment of DNM2 associated with vesicular structures. It is shown a zoom of the vesicles, enlarge in the case of M-DNM2 overexpression, and we can see colocalization of CHC and DNM2 signal in white. Scale bar =20 µm. The size of DNM2 puncta was measured in 3 independent experiments and it is shown at the right. Each dot represents the average of vesicles size in one cell (***P*= 0.0018 by two-tailed Mann Whitney test). (**b-c**) Data are represented as mean values ± SEM. Source data are provided as a Source Data file.

Supplementary Figure 13

a **DNM2 pull down assay with SH3 domain of BIN1**

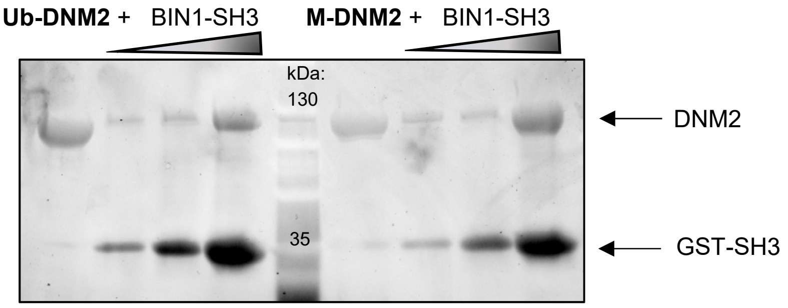

b **SEC-MALS**

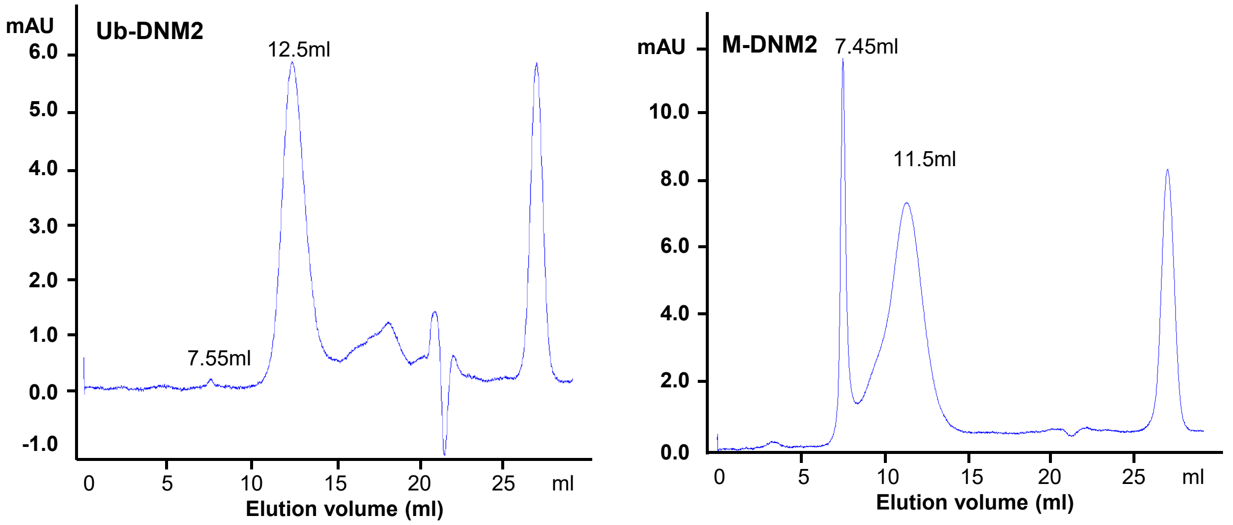

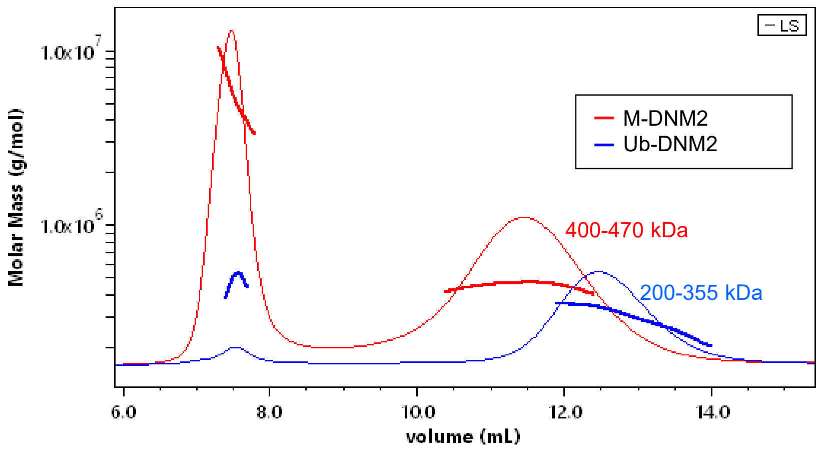

c **Sedimentation assay: depolymerization at high salt**

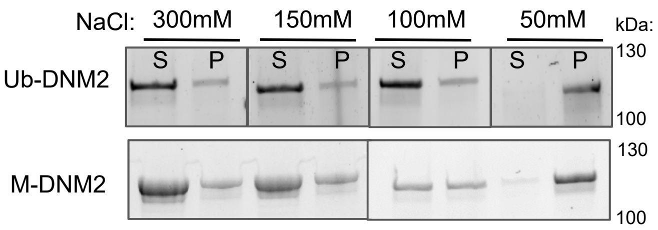

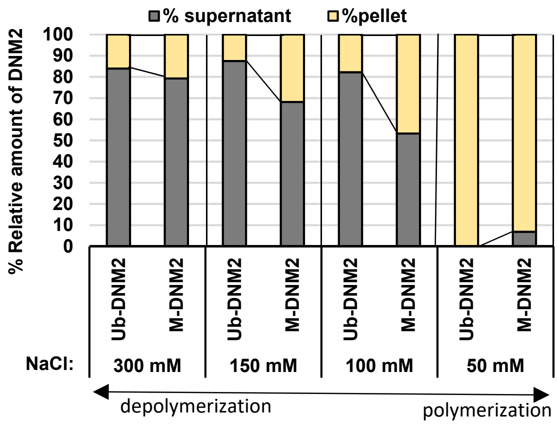

Supplementary Figure 13. M-DNM2 forms higher-ordered oligomers and equally binds BIN1 compared to Ub-DNM2. (**a**) Stain-free images showing dose-dependent binding of M-DNM2 and Ub-DNM2 to the SH3 domain of BIN1. Input: first line of each condition. This result was reproduced in an independent experiment with the same protein. (**b**) Size-exclusion chromatographic profiles of M-DNM2 and Ub-DNM2 run through a Superose 6 10/300 GL column at the same conditions (salt concentration of 1.5 mM NaCl). Protein absorbance at 280 nm (A_{280}) in milli-absorbance units (mAU) is plotted in the graphic as a function of elution volume (ml). Peak obtained with M-DNM2 at 7.45 ml is corresponding to DNM2 aggregates. Below, it is shown the multiangle light scattering (MALS) analysis of molecular mass and indicated the average molecular mass for the main peak. (**c**) Repetition of sedimentation assay at different salt concentrations. It was done with new production of M-DNM2 and Ub-DNM2 recombinant proteins. Stain-free gel is shown, and it was calculated in each case proportion of DNM2 in supernatant (S) and pellet (P). This was reproduced in an independent experiment with another batch of protein production as shown in Fig. 7b.

Supplementary Figure 14

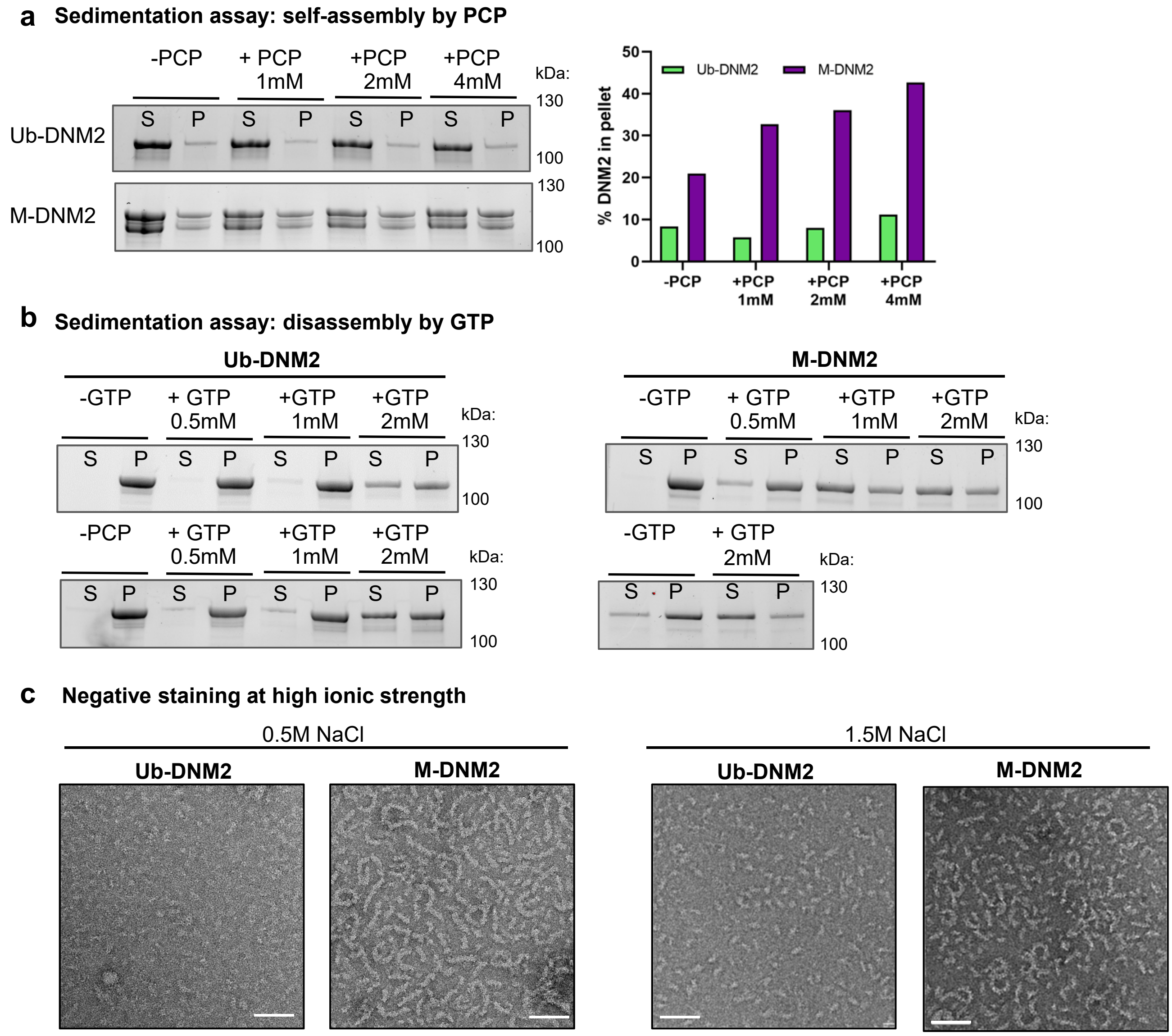

Supplementary Figure 14. Differential oligomerization properties of Ub-DNM2 and M-DNM2 under several conditions. (**a**) Repetition of sedimentation assay at different GMP-PCP (PCP) concentrations. It was done with new production of M-DNM2 and Ub-DNM2 recombinant proteins. Stain-free gel is shown, and it was calculated in each case proportion of DNM2 in supernatant (S) and pellet (P). This result was reproduced in an independent experiment with another batch of protein production as shown in Fig. 7c. (**b**) Stain-free images of 2 more repetitions of sedimentation assay with different concentrations of GTP (0.5 mM GTP + 1 mM $MgCl_2$, 1 mM GTP+ 2 mM $MgCl_2$ and 2 mM GTP+ 4 mM $MgCl_2$). This result was reproduced in an independent experiment with another batch of protein production shown in Fig. 7d and quantification of 2 mM GTP condition is also included in that figure. (**c**) Electron microscopy of M-DNM2 and Ub-DNM2 organization, negative stained, at high salt concentrations. Scale bar=100 nm. Similar result was obtained in an independent experiment with another batch of protein production.

Supplementary Figure 15

a **% GTPase activity, time course**

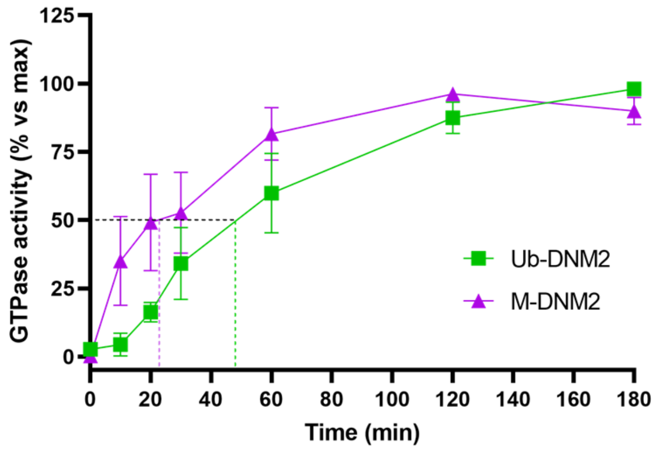

b **Sedimentation assay: depolymerization by GTP, time course**

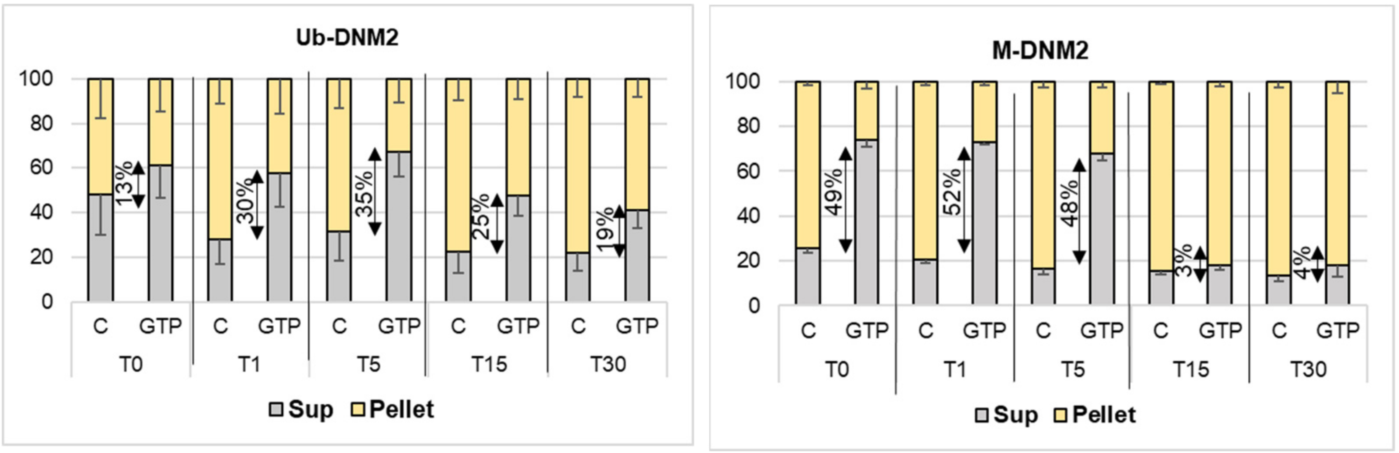

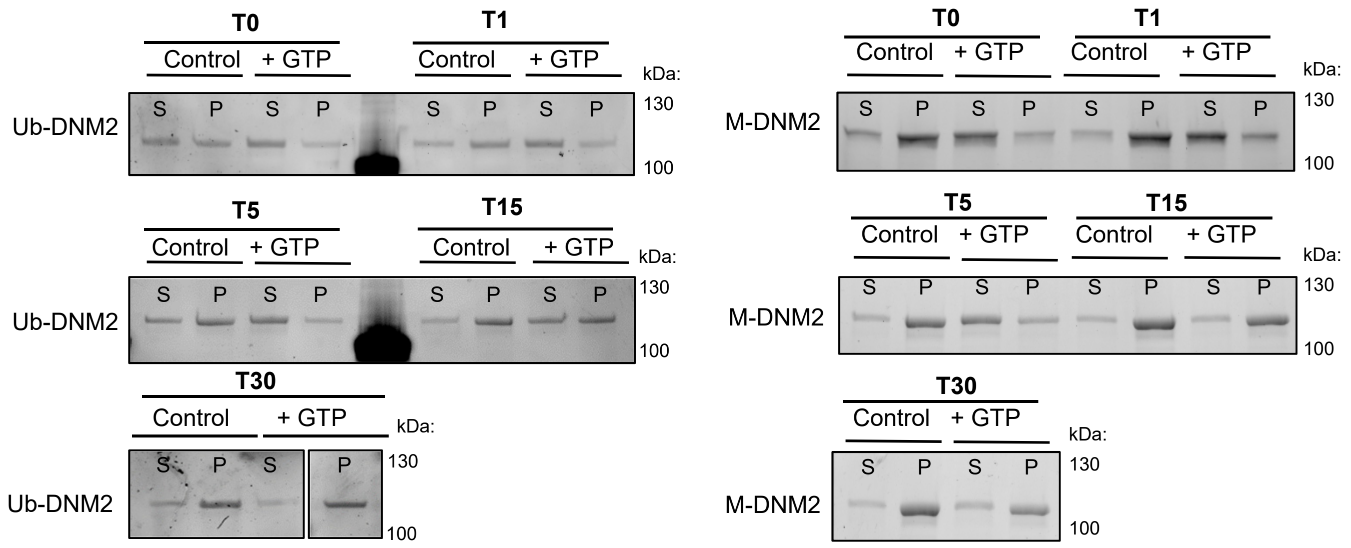

Supplementary Figure 15. Time course of GTP-induced depolymerization. (**a**) Basal GTPase activity at 37.5 mM NaCl and 0.5 mM GTP. % was calculated as the ratio of activity in each time point versus maximum activity achieved (repeated in n=4 independent experiments for Ub-DNM2 and n=5 for M-DNM2 done with proteins coming from 2 different batches of production). Dashed lines indicated time to achieve 50% maximal activity (M-DNM2: ~20 min, Ub-DNM2: ~40 min). (**b**) M-DNM2 and Ub-DNM2 recombinant proteins at 37.5 mM NaCl were incubated with 0.5 mM GTP at different time points (T0, T1, T15, T30), followed by centrifugation and pellet (P) and supernatant (S) fractionation. DNM2 in S and P was quantified from an image of stain-free gel (representative image is shown below). C: control condition without GTP. % indicated in the figure is the difference of DNM2 in pellet between control condition (without GTP) and after incubation with GTP (this percentage is represented in graphic from Figure 7i). Repeated in 3 independent experiments with proteins for 2 different batches of production. (**a, b**) Data are represented as mean values ± SEM. Source data are provided as a Source Data file.

Supplementary Figure 16

Fig. 1e

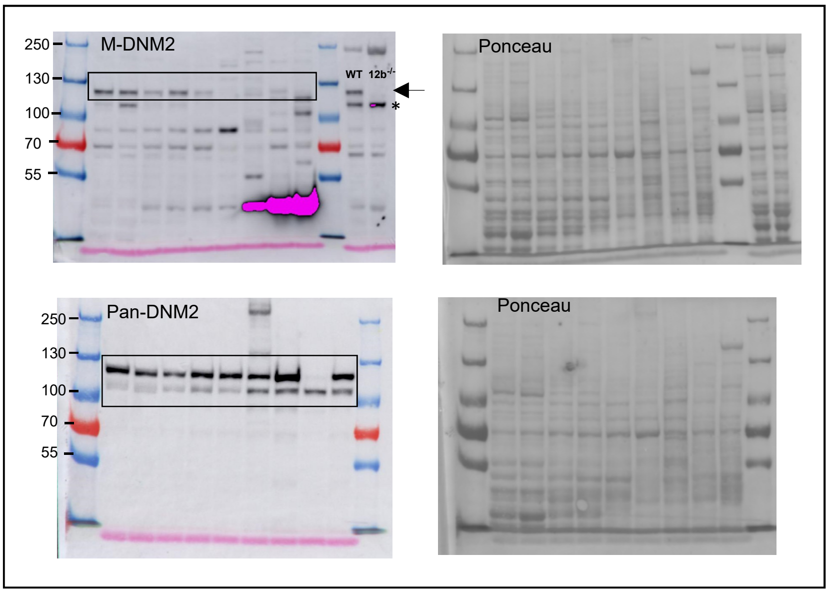

Fig. 3d

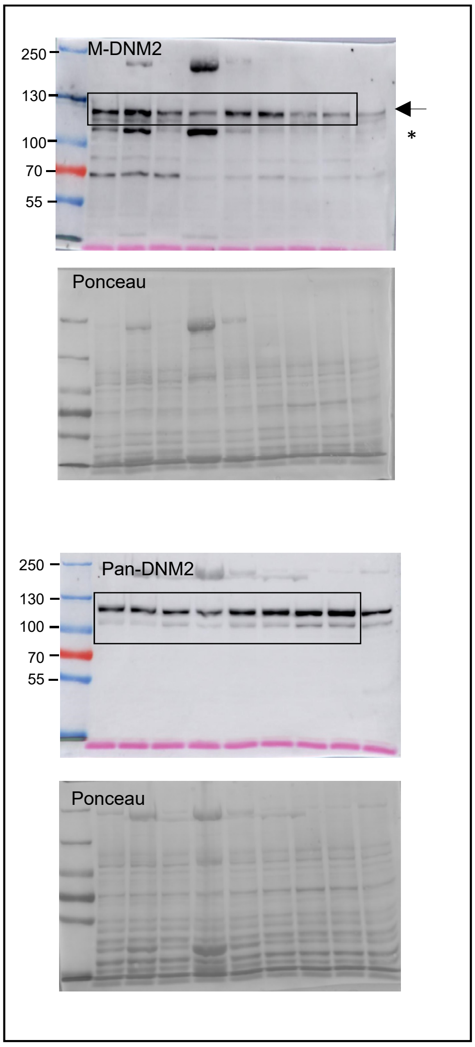

Fig. 2b

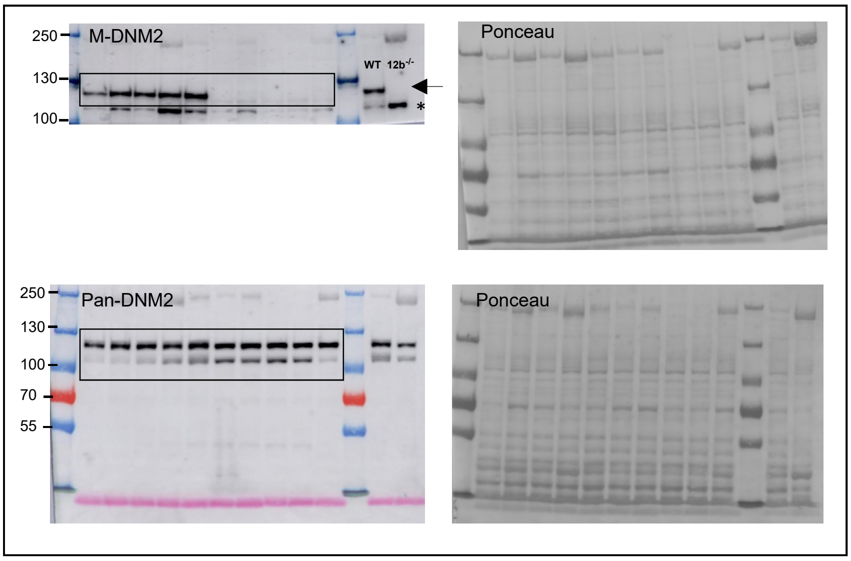

Fig. 4a

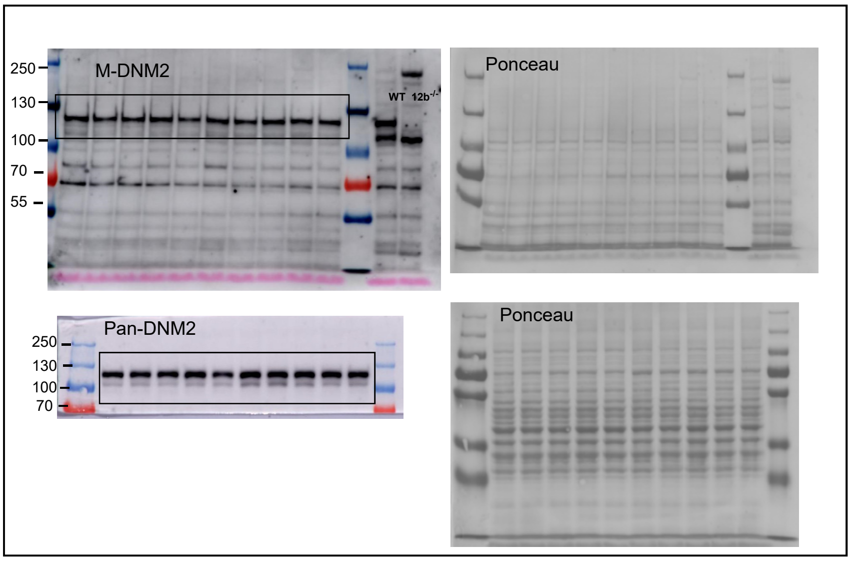

Fig. 5a

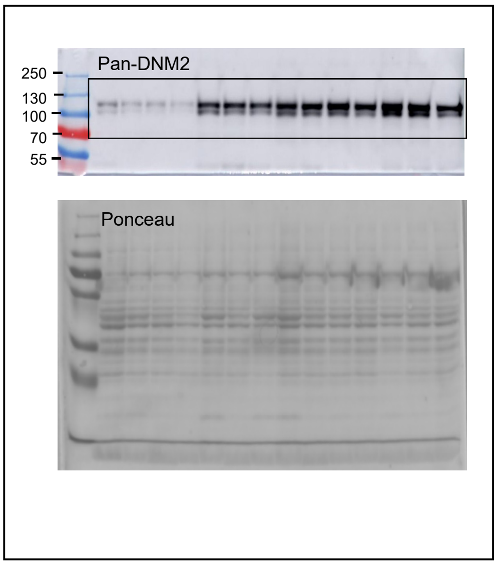

Supplementary Figure 16 Continued

Fig. 6e

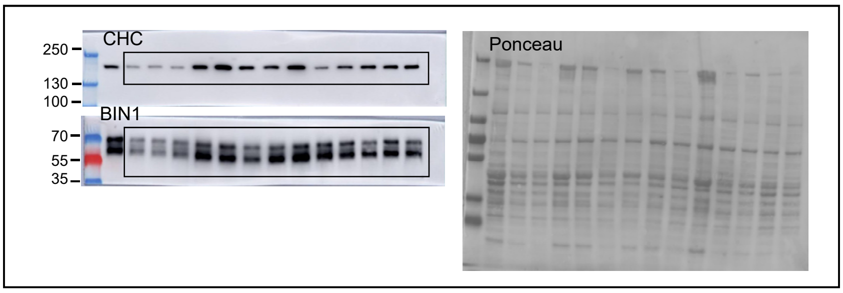

Supplementary Fig. 1d

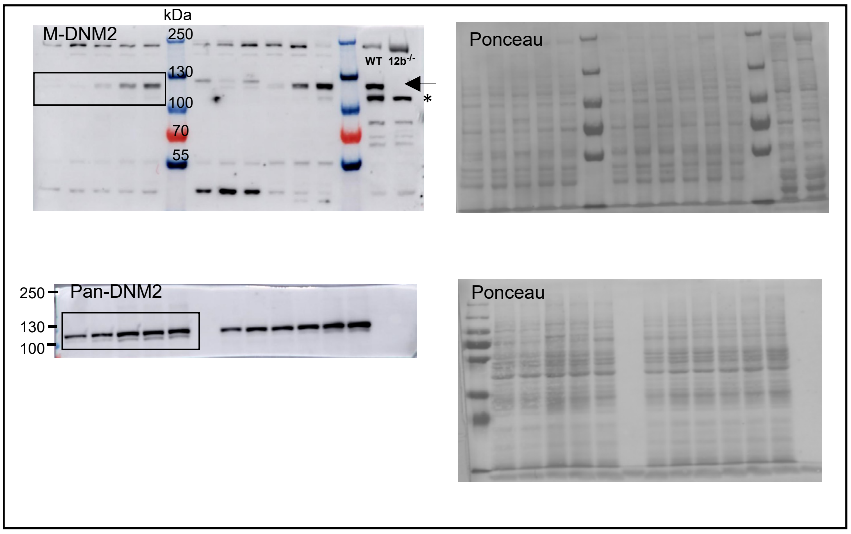

Supplementary Fig. 2c

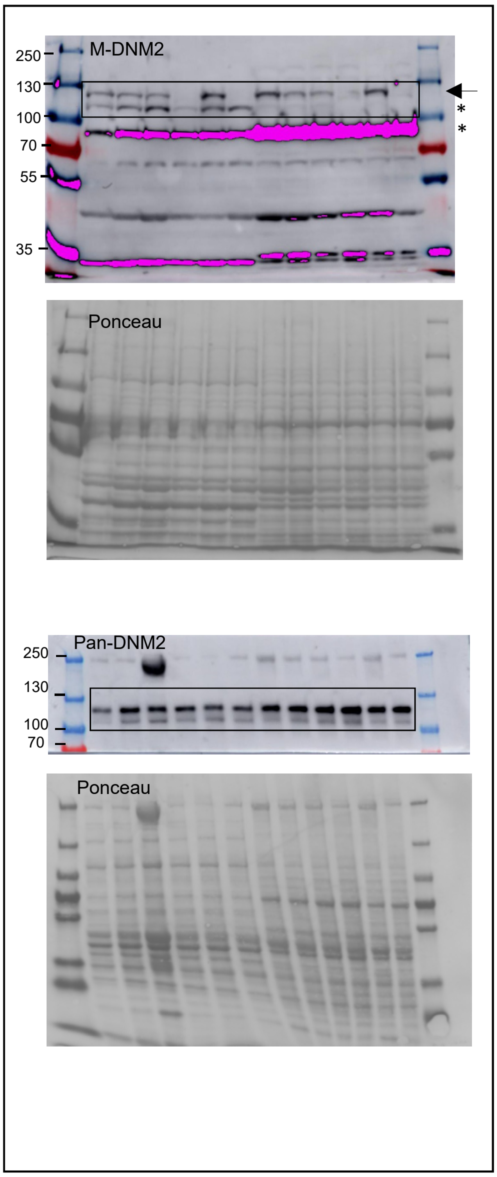

Stain-free gels

Fig. 7b

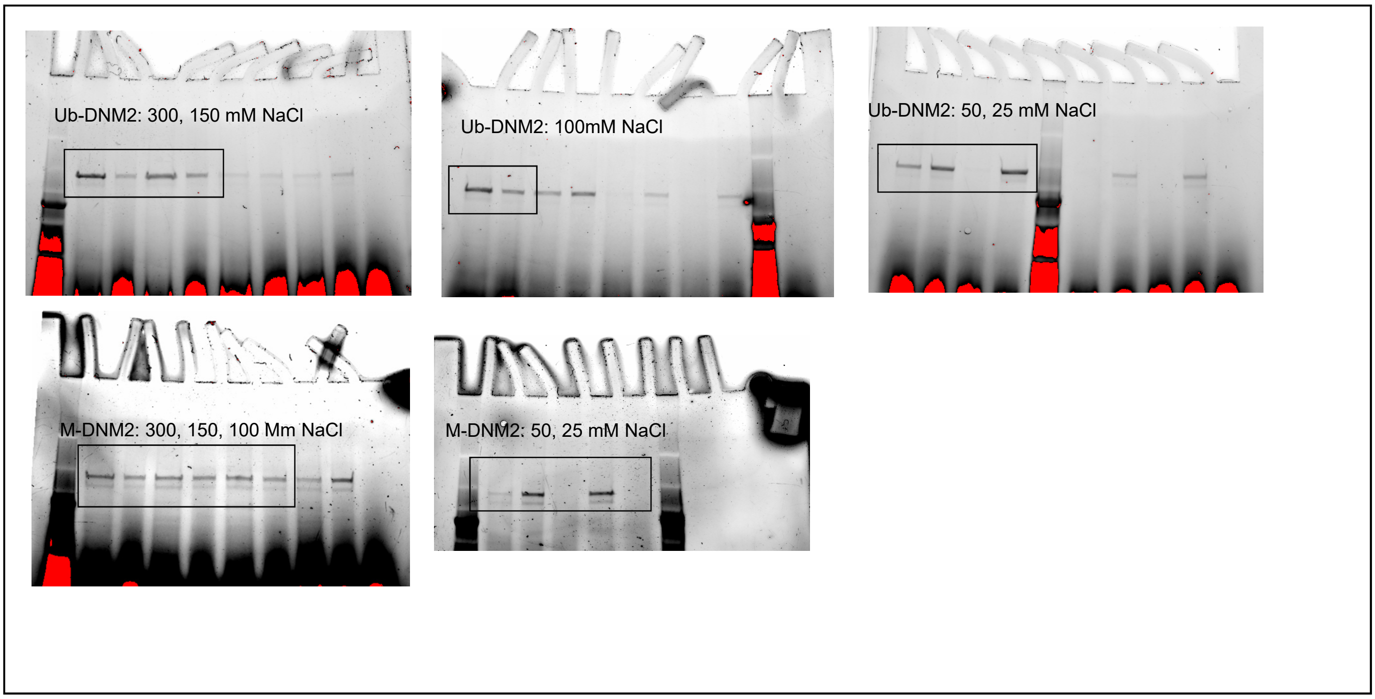

Supplementary Figure 16 Continued

Stain-free gels

Fig. 7c

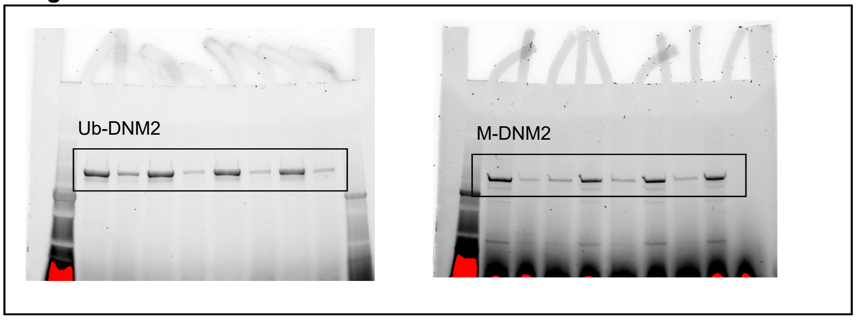

Fig. 7d

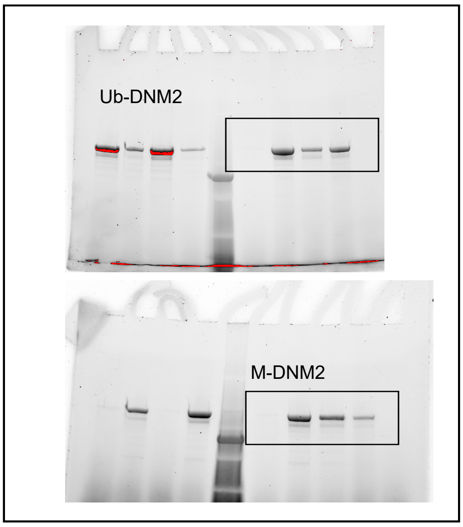

Supplementary Fig. 13c

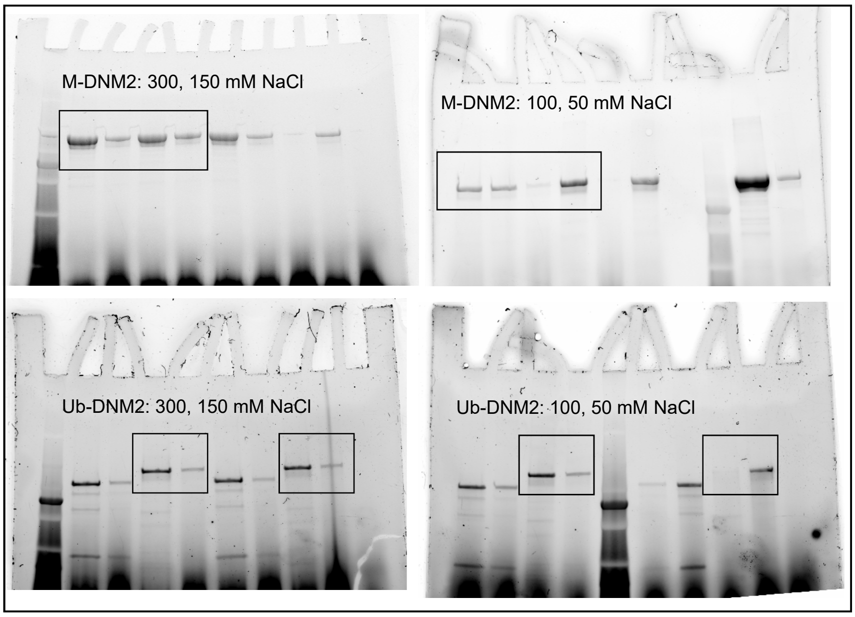

Supplementary Fig. 14a

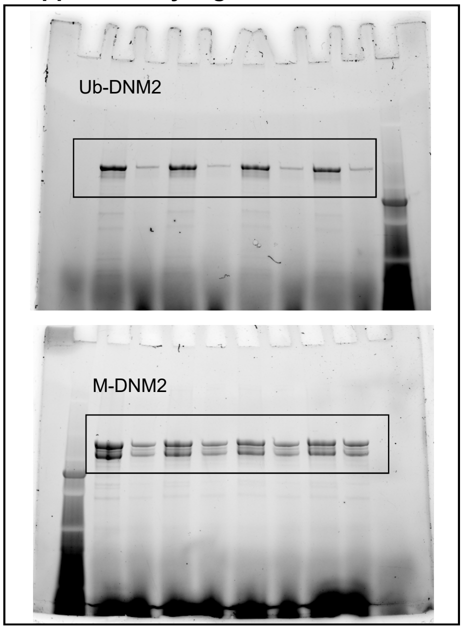

Supplementary Fig. 14b

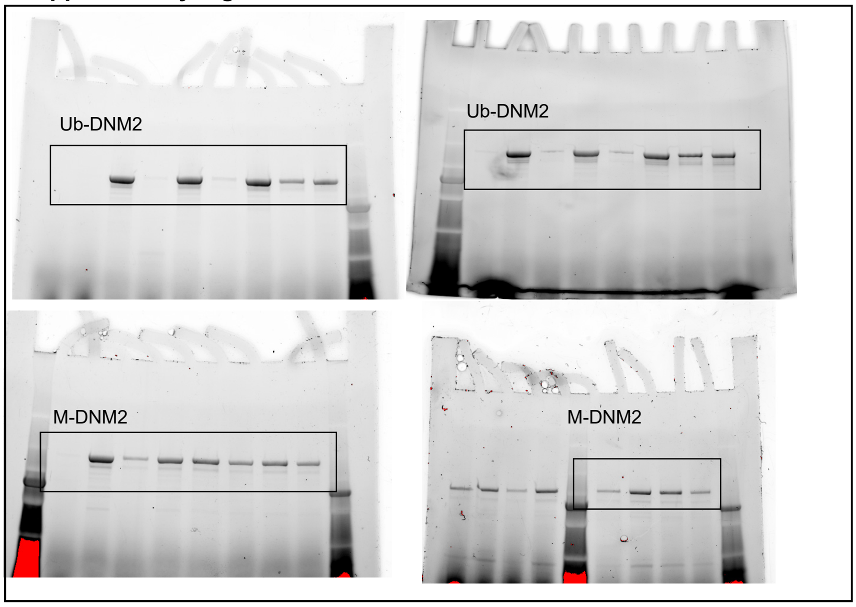

Supplementary Figure 16 Continued

Supplementary Fig. 15b

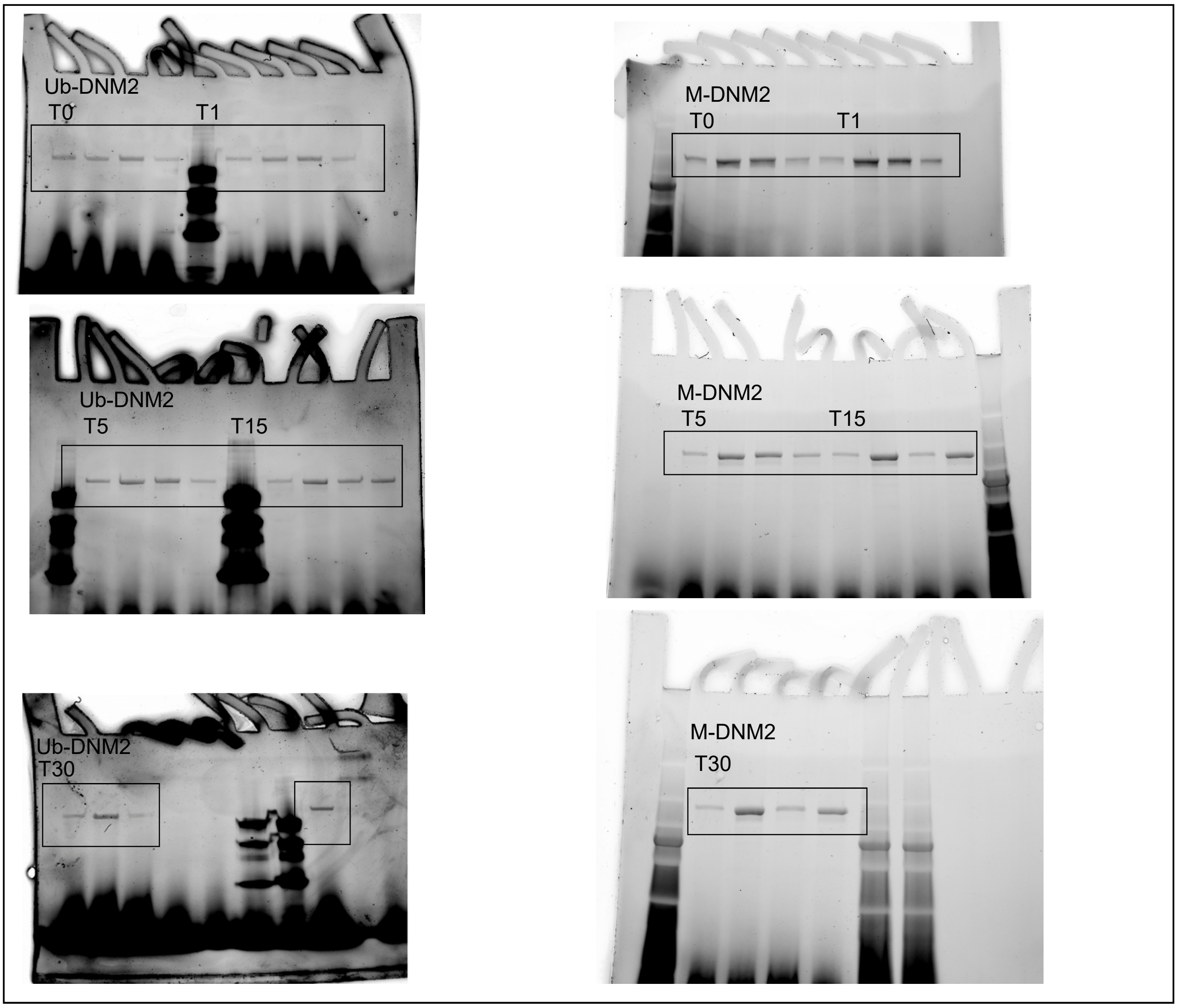

Supplementary Figure 16. Uncropped and unprocessed western blot and Stain-free gel scans showed in main and supplementary figures. Full membrane with protein ladder and molecular weight indicated in kilodalton (kDa). The arrow points to specific band for M-DNM2 antibody and * as unspecific band. Some western blots were run in parallel to muscle samples knock-out for exon 12b (*Dnm2*ex12b$^{-/-}$), as a control.

Supplementary Tables

Supplementary Table 1. Breeding strategy and outcome for *Dnm2*ex12b$^{+/-}$ x *Dnm2*ex12b$^{+/-}$ with expected mice and obtained animals 10 days post-natal.

	Breeding: *Dnm2*ex12b$^{+/-}$ x *Dnm2*ex12b$^{+/-}$			
Outcome	Expected N°	Observed N°	**Expected %**	**Observed %**
**WT**	61	56	**25.00**	**22.95**
***Dnm2*ex12b$^{+/-}$**	122	123	**50.00**	**50.41**
***Dnm2*ex12b$^{-/-}$**	61	65	**25.00**	**26.64**
TOTAL	244.00	244.00	**100.00**	**100.00**

Supplementary Table 2. Summary of tests done to measure locomotor activity and muscle performance of mice at 8 weeks of age

	males + females					
	**WT**		***Dnm2*ex12b -/-**		*P*	Test used
	Mean ±SEM	n	Mean ±-SEM	n		
**Rotarod: Latency to fall (s)**	195.3 ± 11.65	5	200.7 ± 14	6	ns	Two-tailed unpaired t-test
**Grip strenght 4 paws (g/g)**	0.0075 ± 0.0014	6	0.0070 ± 0.0008	6	ns	Two-tailed unpaired t-test
**Actimetry: locomotor activity (photocell counts)**	1040 ± 355.6	6	1262 ± 382.7	5	ns	Two-tailed unpaired t-test

not significant (ns)

Supplementary Table 3. Breeding strategy and outcome for Mtm1$^{+/-}$ x *Dnm2*ex12b$^{+/-}$ with expected mice and obtained animals 10 days post-natal.

	Breeding: *Mtm1*$^{+/-}$ x *Dnm2*ex12b$^{+/-}$			
Outcome	Expected N°	Observed N°	**Expected %**	**Observed %**
**Male WT**	14.25	22	**25.00**	**38.60**
**Male *Dnm2*ex12b$^{+/-}$**	14.25	18	**25.00**	**31.60**
**Male *Mtm1*$^{-/y}$**	14.25	14	**25.00**	**24.60**
**Male *Mtm1*$^{-/y}$ *Dnm2*ex12b$^{+/-}$**	14.25	3	**25.00**	**5.20**
TOTAL	57.00	57.00	**100.00**	**100.00**

	Breeding: *Mtm1*$^{+/-}$ x *Dnm2*ex12b$^{+/-}$			
Outcome	Expected N°	Observed N°	**Expected %**	**Observed %**
**Female WT**	18.00	14	**25.00**	**19.40**
**Female *Dnm2*ex12b$^{+/-}$**	18.00	17	**25.00**	**23.60**
**Female *Mtm1*$^{+/-}$**	18.00	22	**25.00**	**30.60**
**Female *Mtm1*$^{+/-}$ *Dnm2*ex12b$^{+/-}$**	18.00	19	**25.00**	**26.40**
TOTAL	72.00	72.00	**100.00**	**100.00**

Supplementary Table 4. Breeding strategy and outcome for Mtm1$^{+/-}$ *Dnm2*ex12b$^{+/-}$ x *Dnm2*ex12b$^{+/-}$ with expected mice and obtained animals 10 days post-natal.

	Breeding: *Mtm1*$^{+/-}$ *Dnm2*ex12b$^{+/-}$ x Dnm2ex12b$^{+/-}$			
Outcome	Expected N°	Observed N°	**Expected %**	**Observed %**
**Male WT**	15.1	31	**12.50**	**25.6**
**Male *Dnm2*ex12b$^{+/-}$**	30.3	44	**25.00**	**36.4**
**Male *Dnm2*ex12b$^{-/-}$**	15.1	19	**12.50**	**15.7**
**Male *Mtm1*$^{-/y}$**	15.1	21	**12.50**	**17.4**
**Male *Mtm1*$^{-/y}$ *Dnm2*ex12b$^{+/-}$**	30.3	6	**25.00**	**5.0**
**Male *Mtm1*$^{-/y}$ *Dnm2*ex12b$^{-/-}$**	15.1	0	**12.50**	**0**
TOTAL	121.00	121.00	**100.00**	**100.00**

	Breeding: *Mtm1*$^{+/-}$ *Dnm2*ex12b$^{+/-}$ x Dnm2ex12b$^{+/-}$			
Outcome	Expected N°	Observed N°	**Expected %**	**Observed %**
**Female WT**	20.3	19	**12.50**	**11.7**
**Female *Dnm2*ex12b$^{+/-}$**	40.5	45	**25.00**	**27.8**
**Female *Dnm2*ex12b$^{-/-}$**	20.3	20	**12.50**	**12.3**
**Female *Mtm1*$^{+/-}$**	20.3	25	**12.50**	**15.4**
**Female *Mtm1*$^{+/-}$ *Dnm2*ex12b$^{+/-}$**	40.5	42	**25.00**	**25.9**
**Female *Mtm1*$^{+/-}$ *Dnm2*ex12b$^{-/-}$**	20.3	11	**12.50**	**6.8**
TOTAL	162.00	162.00	**100.00**	**100.00**
