# Supplementary material for: Differential impact of ubiquitous and muscle dynamin 2 isoforms in muscle physiology and centronuclear myopathy
Source: Nat Commun. 2022 Nov 11;13:6849. doi: 10.1038/s41467-022-34490-4 (PMC9652393; doi:10.1038/s41467-022-34490-4)

Description of Additional Supplementary Files

Supplementary Movie 1. 4-week-old, WT, *Mtm1*$^{-/y}$, *Mtm1*$^{-/y}$ *Dnm2*ex12b$^{+/-}$ in a cage.

Starting position of the mice for video 1:

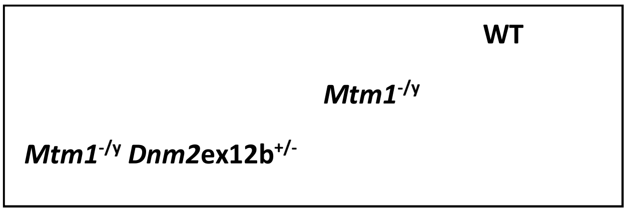

Supplementary Movie 2. 4-week-old, *Dnm2*ex12b$^{+/-}$, *Mtm1*$^{-/y}$, *Mtm1*$^{-/y}$ *Dnm2*ex12b$^{+/-}$ in a cage.

Starting position of the mice for video 2:

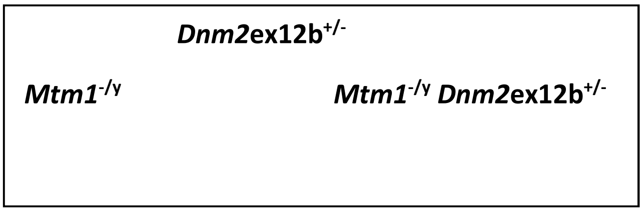

Supplementary Movie 3. 4-week-old *Mtm1*$^{-/y}$, *Mtm1*$^{-/y}$ *Dnm2*ex12b$^{+/-}$ on a grid.

Starting position of the mice for video 3:

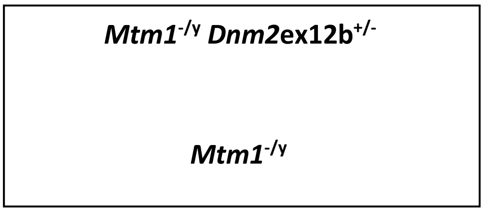

Supplement: Supplementary file 3 — Description of Additional Supplementary Files [file 41467_2022_34490_MOESM3_ESM.pdf]
